# More than one way to improve a CAT: Outcomes and reflections on two iterations of the Queen Square Intensive Comprehensive Aphasia Programme

**DOI:** 10.1080/02687038.2023.2286703

**Published:** 2023-12-06

**Authors:** Alexander Leff, Catherine Doogan, John Bentley, Bani Makkar, Luisa Zenobi-Bird, Amy Sherman, Simon Grobler, Jennifer Crinion

**Affiliations:** a UCL Queen Square Institute of Neurology, University College London, London, UK; b University College London Hospitals NHS Trust, London, UK; c Institute of Cognitive Neuroscience, University College London, London, UK; d Sirona Care & Health, Bristol, UK; e St Georges, University of London

**Keywords:** PWA, ICAP, audit, language, quality of life, mood

## Abstract

**Background:**

The field of human expert performance teaches us that high quality, high-dose guided practice is required to make large gains in cognitively driven acts. The same also seems to be true for people with acquired brain injury, yet therapy services for people with aphasia (PWA) have traditionally not been designed with this in mind. Intensive Comprehensive Aphasia Programmes (ICAPs) are one way to address the chronic under-dosing of therapy that most PWA experience.

**Aims:**

There are several ways to deliver an ICAP; here we describe two iterations of our Queen Square ICAP. There was a 20-month COVID-induced pause between the Year 1 (Y1) and Year 2 (Y2) ICAP groups. We analyse ICAP-induced changes in both groups of PWA on a series of key outcome measures that span the International Classification of Functioning, Disability and Health, covering language impairment and function as well as mood and social participation.

**Methods & Procedures:**

Forty-six PWA took part in Y1 and 44 in Y2. The PWA were all in the chronic stage post stroke and varied in aphasia severity from mild to severe, with the Y2 group being more impaired than Y1. Quantitative data was collected before and after the ICAP. The Y2 therapy team provided independent reflections on their experiences of delivering an ICAP.

**Outcomes & Results:**

ICAP-related changes in outcome measures (impairment, function and goal attainment) were generally comparable for the Y1 and Y2 groups, with both groups’ speech production abilities improving the most. Both groups made clinically and statistically significant gains on the main quality of life measure. Participation in the ICAP made a big difference to PWAs’ self-confidence ratings. Their mood ratings also improved significantly, although they were not, on average, in the depressed range at baseline (directly pre-ICAP). All improvements achieved in both groups were maintained at the 3-month follow-up, highlighting the lasting effects that ICAPs can provide.

**Conclusions:**

Evidence continues to accrue that ICAPs are an efficient way of increasing the dose of expert coaching required for people with chronic aphasia to make clinically meaningful improvements in their communicative abilities and quality of life. The main challenge remaining is convincing health-care providers to invest in them.

## 1. Introduction

“In sum, our empirical investigations and extensive reviews show that the development of expert performance will be primarily constrained by individuals’ engagement in deliberate practice and the quality of the available training resources.” K. Anders Ericsson

Ericsson’s quote relates to people attaining superior performance in sports, the arts and professions (Ericsson et al., 2009), but doesn’t this statement equally apply to people recovering from brain injury? We think so. The evidence from meta-analyses of interventional studies in people with aphasia (PWA) certainly highlights the importance of dose required to make clinically meaningful changes in their functional communication. Bhogal’s seminal meta-analysis suggested 100 hours (Bhogal et al., 2003), the latest Cochrane review between 60 and 208 hours (Brady et al., 2016) and the most recent evidence from the RELEASE project, 50+ hours (Brady et al., 2022). This begs the question, how can PWA achieve high enough doses of contact with expert coaches (therapists) that will make a difference to them? While the evidence from pedagogy and some aphasia studies suggests that spacing out practice may be best (J. K. Dignam et al., 2015), economic factors have driven the rise of ICAPs as a way of providing large doses over short time scales. A therapy team working on a single site is more efficient time-wise than the peripatetic solo therapist in treating cohorted groups of PWA. The aim is for the ICAP to produce a big enough step-change in language function to enable PWA to engage more with others and thus maintain or even boost any ICAP-related gains that are made over a short period of time. Minimum numbers of contact hours for a service to be considered as an ICAP are three hours a day for two weeks (Rose et al., 2022), so >30 hours in total; this is considerably more than most community-based PWA will ever receive (Code & Heron, 2003). To date, nine ICAPs that have treated ten or more patients have published their findings, all have employed a pre-post analysis using a variety of standardised outcome measures. We have summarised their key attributes, including effect sizes on impairment and function-based outcome measures, in Table 1. In short, most have demonstrated medium to large effects on key language-based outcome measures with only two bucking this trend (Griffin-Musick et al., 2022; Winans-Mitrik et al., 2014).

**Table 1:** Summary of published ICAP studies to date (with a minimum of 10 PWA reported) showing key variables and effect sizes summaries of pre-post analyses on impairment and function outcomes. ^a^ = proportion of participants who achieved a clinically significant outcome, ^b^ = calculated from data presented in the paper and estimated using a standard Cohen’s *d* analysis (i.e. between group), N = number of PWA, Nil = no significant effect size, NR = not reported.

ICAP	1st Author	Year	N	Time post onset (months)	Duration (weeks)	Dose (hours)	Outcome effect sizes	
							Impairment	Function
LIFT	Rodriguez	2013	11	23	2-4	40-100	Nil	large
UMAP	Persad	2013	54	16.5	6	138	77%^a^	NR
InteRACT	Persad	2013	70	24.5	4.5	112	64%^a^	51%^a^
PIRATE	Winans-Mitrik	2014	73	18	4.5	115	small^b^	small^b^
RIC-UQ	Babbitt	2015	74	15.5	4	120	large	large
Boston	Hoover	2017	27	59	4	120	large	medium^b^
Queen Square	Leff	2021	47	29	3	90	large	large
Big Sky	Griffin-Musick	2021/22	37/48	60/34	3-5	36-72	small	medium
S-IHP	Nicholas	2022	35	34	6	150	large	large

The Queen Square ICAP was established to help PWA receive high dose intervention, contribute to the evidence-base, and build a case for commissioning. The funding model was mixed. The clinical space and line-management team were provided by the NHS funded hospital (National Hospital for Neurology and Neurosurgery, part of University College London Hospitals NHS Foundation Trust) and the ‘on costs’ (~90% staff costs) were provided by a charity, The National Brain Appeal (£600,000 over two years).

Our approach in the first year of the Queen Square ICAP (Y1) was to aim for a high dose (Bhogal’s 100 hours) over a three-week period with cohorts of four PWA attending Monday to Friday with a full, all-day (seven-hour) timetable. We got close to this, ~90 hours on average. While PWA seemed to tolerate this (one dropout out of 46 PWA), it took its toll on the therapists who did not have enough non-contact time to manage their administrative duties (e.g., note keeping, session planning, team meetings, onward referrals). We timetabled a ‘reflection week’ once a quarter with the therapy team choosing the content (e.g., sessions on data analysis, academic learning and outputs, reflective-practice, and changes to service delivery) but quite quickly, these weeks were used to ‘catch-up’ on administrative tasks. By the time the service was halted due to the COVID-19 pandemic (March 2020) most of the treating team reported a degree of burn-out.

20 months later, we were able to restart the service in January 2022 (Y2). The COVID enforced pause enabled us to have frank discussions with service provision stakeholders and make some key structural and implementation changes (Table 2). Perhaps most importantly, we changed the site of the ICAP, which had been running on our inpatient Neuro Rehabilitation Unit, where space was extremely limited, to a separate outpatient facility a ~20 min walk away from the main hospital site. This gave us more workspace, with bookable clinic and meeting rooms (although the space was still shared with another clinical service). Secondly, we altered the team line-management structure. Previously the senior speech and language therapist (SLT) (UK NHS, band 8b) both line-managed the team and took part in delivering parts of the ICAP service. In Y2 we split the roles with a senior SLT (SG) who provided direct line-management to the team, including clinical supervision, but did not treat PWA on the ICAP. We also gave the treating team a more hierarchical structure with a senior specialist SLT, UK NHS band 8a (JB) as the leading therapist with overall management provided by the clinical academics (APL and JC) having weekly ward-round meetings with the therapy team and quarterly strategic review meetings with the line-manager. Lastly, while the Clinical Psychologist continued to provide support to the PWA cohorts (running a weekly adjustment group), they also set up a bespoke group therapy service for their carers (a weekly carers’ café) that ran both during the ICAP and after the PWA had finished the ICAP programme.

**Table 2:** Main structural differences between the two ICAP year groups in terms of the composition of the treating team, PWA cohorting, site and main psychological interventions. B = Band (high numbers = more experienced posts), WTE = whole time equivalent.

Year	Therapy Team	Model	Site	Psychology
Y1	0.1 WTE B8b SLT 2.0 WTE B7 SLT 2.0 WTE B3 Rehab Assistant 0.5 WTE B7 Psychologist 0.5 WTE B4 Admin	4 PWA 5 days a week 3 weeks = 15 days 46 PWA (1 drop-out)	Space shared with in-patient service	Narrative therapy group (PWA) Family and friends support group (carers) Couple/family therapy interventions (PWA and carers)
Y2	1.0 WTE B8a SLT 1.0 WTE B7 SLT 1.0 WTE B6 SLT 1.0 WTE B5 SLT 0.4 WTE B8b Psychologist 0.5 WTE B4 Admin	4 PWA 4 days a week 4 weeks = 16 days 44 PWA (0 drop-outs)	Dedicated out-patient space	Meet the Drs (PWA and carers) Adjustment group (PWA) Carers’ Café (carers)

The aims of this paper are to outline the key operational elements of the Y2 ICAP, present the quantitative results, provide individual reflective narratives from the treating team and make suggestions for future iterations of ICAPs.

## 2. Materials and methods

### 2.1. Key ICAP elements

A full operating manual for the Queen Square ICAP is available as a supplement to this article. The key components will be summarised here.

#### 2.1.1. Weekly structure

The Y2 model allocated four days of the week to direct clinical treatment and preserved a single working day (Monday) for interdisciplinary discussion, planning, resource development, pre-attendance assessment and general administration duties. A weekly ward round involving all team members (except the administrator who attended monthly) took place in the morning of the non-clinical day. Each PWA was allocated a keyworker on admission to the service. The keyworker acted as the primary point of contact for the participant and their family and completed the individual’s baseline assessment where possible. The key worker and Clinical Psychologist would present the joint formulation from the SLT’s initial assessment of the PWA and the Clinical Psychologist’s assessment of the close other (carer). As the PWA moved through the programme the PWA and carers were reviewed with reference to how they were experiencing the ICAP, their therapeutic priorities and goal achievement. We also discussed the planning structure and scheduling of the programme, familiarisation with forthcoming cohort participants and any administrative challenges. As the cohorts moved through the ICAP, we responded to both their feedback to reshape parts of the programme.

#### 2.1.2. Timing of assessments and documentation

We scheduled the pre-admission baseline assessment (both language and psychology) a few weeks before admission to the programme. Carers/significant others were invited to attend as well. Three hours were allocated for orientation, baseline assessment and scoring. The end of ICAP assessments occurred on the penultimate or final day of the ICAP (i.e., treating day 15 or 16). This arrangement reduced the pressure on staffing and was viewed as optimal from a participant performance perspective (e.g., avoided after-lunch fatigue). Two hours were allocated for final assessment and scoring. We scheduled the 3-month, post-ICAP follow-up assessment on Monday (the non-clinical day); again, three hours were allocated for this assessment and scoring. Documentation demands were kept to a minimum given that treating clinicians were required to deliver therapy for >6 hours a day. All therapy session notes were written into each participant’s individual EXCEL recording document (stored in their individual folders) within 24 hours of the session or activity. These were then uploaded to our hospital’s electronic patient records system. The carer’s assessment contributed to the systemic psychological formulation of the PWA and considered the carer’s experience and their goals.

#### 2.1.3. Regular group therapeutic activities

Daily timetables were structured to combine individual treatments, paired activities and whole group therapies that targeted specific impairments, improved use of appropriate strategies and positive functional change. Almost all individual and paired sessions were tailored to the specific needs of participants whereas group activities (selected in accordance with the needs and preferences of the group) were often more geared to supporting generalisation of skills and strategies into naturalistic communication. Schedules were structured in such a way as to limit fatigue and typically each morning and afternoon session would adhere to the same format; beginning with a moderately challenging group session followed by a social tea-break ahead of extended individual or paired work at a higher intensity before rounding off with a lighter, more enjoyable group session ahead of lunch/departure. The following descriptions help to illustrate the aims and breadth of activity that regularly featured in whole group sessions:

1. **Introduction Group**: Assess participants’ level of abilities to provide information about themselves, with and without support, and to help build group rapport and familiarity.
2. **Tea Break**: Develop participants’ ability to initiate requests for objects and actions (i.e., ‘a cup of tea’ or ‘put two sugars in it’) with attention to all necessary information using either a) a fixed phrase that becomes established beyond rehearsal and practical application or b) mixed phrases (“can I have … ” “I would like … ” “could you get me … ”) used interchangeably. As well as opportunities for spontaneous conversations and getting to know each other better.
3. **What Is Aphasia**: Allow participants to describe their individual experiences of stroke and aphasia. This occurred on the first day of each ICAP cohort.
4. **Aphasia Education**: Optimise systemic communication, for PWA to better understand their condition, what helps to promote improvement/what hinders further recovery and to communicate this learning to selected people in their system (family, friends, colleagues etc.).
5. **Verb PACE (promoting aphasic communication effectiveness)**: Establish the value of imitating actions not only as a useful compensatory behaviour but as a stimulus to spoken verb retrieval and to develop use of gesture assisted lexical access as a default behaviour when struggling to express a message.
6. **Newspaper Group**: Increase reading aloud and reading comprehension skills, using stories that were chosen by individual group members.
7. **Annotation Game**: Encourage ideation and spoken naming within each groups’ shared knowledge of a concept and its extrinsic and intrinsic components. Promotes divergent and generative thinking as well as category-based generative naming.
8. **Debating Group**: Develop divergent thinking and reasoning skills using often controversial and emotive topics to elicit discussion. The task required each cohort to be divided into two pairs: one directed to thinking of reasons for a given argument and another thinking of reasons against the same argument.
9. **Self-Directed Therapy**: Develop participants’ independence accessing and progressing with appropriate technological therapy apps using Cuespeak, Tactus Language, Advanced Language, and Conversation bundles, and UCL Listen-In, iReadMore, and iTalkBetter therapy apps. Here PWA used their own tablet/phone with ICAP devices provided (on loan) to those without.
10. **Intensive Language Action Therapy (ILAT)**: Action spoken language use into a range of social communicative contexts using a screen/ barrier to vision as a means of physical constraint, placing all communicative demands on spoken language and auditory comprehension.
11. **Museum Visit**: Develop group cohesion and provide stimulating experiences that inspire detailed descriptions of objects or events (using advised strategies to maximize independent communication) whilst enjoying local museums and exhibits.
12. **Pronoun Games**: Develop participants’ ability to use personal pronouns (especially once an individual or object has been named in the first instance and is thereafter appropriately referred to via pronoun) along with developing awareness and appropriate use of possessive pronouns.
13. **20 Questions**: Develop logical inquiry derived from existing knowledge and awareness of high value versus low value information (salience and relevance). This task uses pictures of many well-known, varied and often contemporary celebrities of different genders, nationalities, ethnicities, and backgrounds. Participants select a celebrity and then field closed questions from the other members of the group who try to guess who it is.
14. **TV or Film Clip ‘Dynamic Description’**: Develop participants’ abilities to perceive the core event in a short film sequence and describe the activity as fully as possible –this also increases awareness of predicate argument structure and thematic role assignment.
15. **Weekend Review**: Encourage all participants to offer as much detail about their weekend activities as possible without direct questioning. Use of smartphone cameras, supportive materials and a total communication approach was encouraged.
16. **Recipe Group**: Aid step-by-step ideation and structured verbal description of a familiar recipe from each participant within the cohort.
17. **Travel Stories Group**: Elicit high quality and stimulating narratives about positive and negative travel experiences using discourse strategies learned in individual therapy.
18. **Process Descriptions Group**: Develop ideation skills in the conceptualisation and description of familiar multi-step tasks (e.g., changing a tyre on a car/making a cup of tea/travelling through an airport).
19. **Music Group**: Share enthusiasm for and knowledge of artists and/or musical works via supported independent research, preparation and rehearsal of key words, phrases or sentence/paragraph level description.
20. **Presentations**: Develop participants’ ability to tell personal narratives and share their identities through personal stories and interests.

*The following three group activities were led by the clinical psychologist*:

1. **Meet The Doctors**: A one-hour open discussion/psycho-educational session with the Clinical Psychologist and the Consultant Neurologist on a variety of topics relating to stroke (e.g., causes, prognosis and prevention) and rehabilitation (e.g., recovery trajectories, plasticity).
2. **Adjustment Group** (facilitated by Clinical Psychologist with SLT support): This activity was to address what life was like before their stroke, getting to know the ‘person’ in order to create a picture of their identity before any changes. This helped to access their values, and established ways of working towards living out this values. We also explored social support and isolation and ways in which to feel more engaged and less lonely.
3. **Carers’ Café**, (supported by psychology assistant or research assistant): Clinical psychology led forum to support the nominated carers of PWA, in order to adjust and adapt to the changes they are experiencing. The group follows the principles of Acceptance and Commitment Therapy (Curvis & Methley, 2021). Themes include letting go of the struggle, increasing mental flexibility, unhooking from the pain and loss, and acknowledging the strain that sometimes accompanies this role.

### 2.2. PWA

Forty-six PWA took part in Y1 of the ICAP and 44 in Y2. There was a single dropout in Y1. PWA were reviewed in an NHS assessment clinic (JC). Inclusion criteria for the ICAP were: chronic aphasia (>3 months post-onset) caused by an acquired brain injury, age >18 years, fluent in English pre-stroke, able to repeat monosyllabic words and a minimum naming score of three on the naming subtest of the CAT. Exclusion criteria were: the presence of severe speech apraxia (no meaningful spoken word output), PWA who were not independent with activities of daily living, PWA with severe reported fatigue or other practical reasons why they could not commit to the timing and duration of the ICAP service. Ongoing psychiatric disorders (e.g., psychosis) were a further exclusion criterion but low mood was not. ~50% of those assessed were deemed suitable to be offered a place on the ICAP. Demographic and baseline behavioural scores for both years’ participants are summarised in Table 3.

**Table 3:** Key baseline variables by PWA Group. Y1 = year 1 cohort, Y2 = year 2 cohort. Values are either medians [IQR], means (SD) or percentages (%). Significant Group differences are in italics. *main effect of Group across all four language domains, see text.

Variable	Y1	Y2	p
Age	50.5 [15]	54.5 [16]	0.631
Gender	70% M	63% M	0.551
Time post onset	28.5 [36]	40 [44]	*0.006*
CAT: Comp	49 (12)	44 (11)	< 0.001*
CAT: Read	45 (12)	29 (24)	
CAT: SPD	14 (11)	12 (10)	
CAT: WPD	8 (10)	6 (6)	

The Queen Square ICAP service is registered as a service audit (National Hospital for Neurology and Neurosurgery: Ref 61-202021-CA), and as such did not require formal ethical approval and the relevant board have waived the need for patient consent for data analysis and publication. However, patient confidentiality was ensured during data collection and data analysis, so that no personal information is identifiable to those outside of the study. Additionally, data was stored securely in encrypted databases which were only shared with researchers and clinicians directly involved with the ICAP.

### 2.3. Outcome measures and quantitative data analysis

The team collected behavioural outcome measures across the range of the International Classification of Functioning, Disability and Health. Some measures were assessed by the treating team while others relied on either patient report (patient reported outcome measures – PROMs), or carer report (carer reported outcome measures – CROMs).

For the PWA-centred outcomes, we report results in the following order: The Comprehensive Aphasia Test (CAT), (Swinburn et al., 2004); The Communicative Effectiveness Index (CETI), CROM (Lomas et al., 1989); Stroke and Aphasia Quality of Life Scale (SAQoL), PROM (Hilari et al., 2003); Stroke Aphasic Depression Questionnaire (SADQ), PROM (Sutcliffe & Lincoln, 1998); Communication Confidence Rating Scale for Aphasia (CCRSA), PROM (E. M. Babbitt et al., 2011). We also collected PWA-specific outcome measures using the goal attainment scale (GAS) (Turner-Stokes et al., 2009) and report short-term (over the course of the ICAP) and medium-term (up to 3-months post ICAP) goals.

For the Carer-centred outcomes (all CROMs), we report the following: Adult Carers Quality of Life questionnaire (ACQOL), (Joseph et al., 2012); the Bakas Caregiving Outcomes Scale (BCOS-10 item version) (Bakas & Champion, 1999); The Hospital Anxiety and Depression Scale (HADS) (Zigmond & Snaith, 1983); Oberst Caregiving Burden Scale (OCBS) (Bakas et al., 2004); The General Self-Efficacy Scale (GSE) (Luszczynska et al., 2005).

#### 2.3.1 Analysis of baseline data

Demographic (age, gender and time since onset) and baseline language behavioural data (CAT), as mentioned in the previous section, were compared between the two ICAP year groups to investigate whether there any significant differences between them. First, the data were visually inspected and subjected to tests of normality (Shapiro-Wilk) to determine whether a parametric (MANOVA or independent-samples t-test) or non-parametric (independent-samples Mann-Whitney U test) be used. Categorical data (gender) were examined using a Chi-Square test. The results are displayed in Table 3.

#### 2.3.2 Analysis of repeated-measures data

The PWA dataset was analysed using IBM Statistical Package for the Social Sciences (SPSS) v.28.0. We used parametric tests to investigate the effects of the ICAP intervention on each set of outcome measures. We ran three types of analyses. Firstly, for those outcomes already published for Y1 (CAT, CETI and GAS scores), we conducted the same analysis for the Y2 participants only, to see if the results were replicable, before completing a comparison of effect sizes between the two groups. Secondly, for all the outcome measures collected across both years but not previously reported (SAQoL, SADQ and CCSRA), we analysed both groups’ data together with Group (Y1 vs. Y2) entered as a between-subject variable. We report Time*Group interactions first, then whether there was a main effect of Time. Lastly, for the carer-reported measures (CROMs), we had planned a repeated measures analysis; however, not enough data was returned at the end of the Y2 ICAP (13/44 = 30%) to usefully report this, so we only report the baseline data (23/44 = 52% of the sample). The main reasons for the low response rate were: the Clinical Psychologist was not in place at the beginning of Y2; they worked part-time, limiting flexibility of scheduling; and, most of the carers were of working age and some had dependents, which was a barrier to joining the carers café which was in person for the duration of the ICAP and remote after the 4 weeks were finished.

For the CAT data we ran a 4*3 repeated-measures MANOVA: Domain as the four language dependent variables (spoken picture description, written picture description, spoken language comprehension total and reading total CAT scores) and Time as a factor with three levels, corresponding to the three timepoints (baseline, directly post-ICAP and 3-month follow-up). The SAQoL was collected at two time points (baseline and 3-month follow-up) and comprises of three domains (physical, psychosocial and communication). For brevity of data capture, we removed the physical domain so our analysis was a MANOVA with Domain as the two dependent variables (psychosocial and communication) and Time as a two-level factor. All the other outcome measures had no sub-domains and were only collected at two time points (baseline and 3-month follow-up), so were analysed using a one-way ANOVA with Group as a between-subject variable. The p-value used to determine significant change was set at the conventional cut-off of <0.05 with Greenhouse-Geisser corrections applied when the data violated tests of sphericity.

We report standardised effect sizes as calculated in SPSS (partial eta squared, where >0.01 is considered a small, >0.06 is considered a medium and >0.14 a large effect size; and, Cohen’s *d* where >0.2 is considered a small, >0.5 is considered a medium and >0.8 a large effect size). Also, given the heterogeneity in PWA’s response to therapeutic interventions, group-level data or average responses can miss important variation in individual outcomes (Menahemi-Falkov et al., 2022). Where published data is available (CETI and SAQoL), we also report the Minimal Detectable Change 90% (MDC_90_), that is, the percentage of PWA who improved by a big enough margin to make it most likely that the change score represents a “true change” and not one that is best explained by measurement error or chance.

### 2.4. Therapeutic approaches for Y1 and Y2

A multidisciplinary team consisting of highly specialised SLTs, SLT assistants, a clinical neuropsychologist and trainee clinical psychologist, and a consultant neurologist provided the ICAP interventions across both years. The content involved patient and family education, individual goal setting, impairment-based therapy (language), communication partner training and facilitative strategies for communications. The interventions were delivered through a variety of approaches, including individual and paired (dyad) sessions (lasting 1-1.5 hours, several per day), group sessions, independent practice (including using therapy apps) and sessions with the patient and their family members. PWA were grouped into roughly-matched cohorts of four participants (based mainly on overall aphasia severity) and were ‘day attenders’ (meaning that they were not resident, but came each day from either their home or a nearby hotel) at the Queen Square ICAP which in Y1 ran for three weeks 09:00-17:00 Monday to Friday and in Y2 four weeks 09:00-17:00 Tuesday to Friday. A random sample of six patients’ (13% of the total from Y2) therapy timetables revealed an average of 95 hours of therapy (range 88-100). This included SLT and Psychology groups with friends and family (~10% of therapy time). A full description of the ICAP intervention for Y1 is presented as supplemental material in a prior paper (Leff et al., 2021) including TIDieR guidelines of the service and details on the types of therapy sessions provided and staff: PWA ratios for them. The Y2 team produced an Operating Manual which contains all this and more (e.g., sample weekly timetables) and is provided as an appendix to this paper.

## 3 Results

### 3.1. Baseline comparison Y1 vs. Y2

There were no significant differences between the two groups in terms of age or gender, but Y2 had a significantly greater time since stroke onset than Y1 by an average of 11.5 months U = 1354.5, *p* = .006. A MANOVA demonstrated that Y2 were also significantly more language impaired than Y1 across the four domains of the CAT, *F*(4, 84) = 5.29, *p* = <.001. Y2 had numerically smaller scores for all domains (Table 3), with the group effect mainly being driven by differences in the language perception domains (reading *F*(1, 87) = 15.05, *p* = <.001, and comprehension *F*(1, 87) = 3.75, *p* = .056).

### 3.2. Replication of previously published outcomes using Y2 data only

#### 3.2.1. CAT: baseline/post-ICAP/3-month follow-up

**Figure 1:**
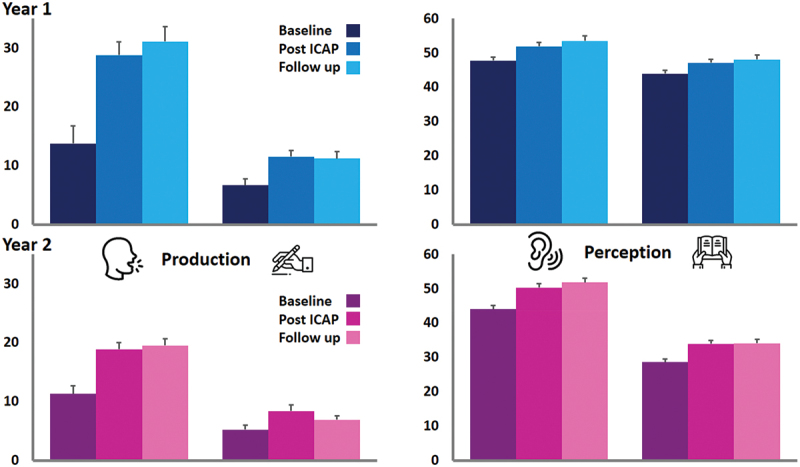
Mean CAT outcome scores for the four main language domains (production = speaking and writing, perception = auditory comprehension and reading) across the three time points (Baseline, immediately Post ICAP and 3-month follow-up), for the two Groups (Year 1 = blue, Year 2 = pink). Error bars are within-subject 95% confidence intervals.

A previously published analyses of Y1 data across the three time points revealed a Time*Domain interaction with scores on SPD improving significantly more than the other three domains (which also all significantly improved). The identical MANOVA analysis was applied to the Y2 data which also revealed a Time*Domain interaction *F*(6, 252) = 5.94, p = <.001; however, this was driven by significantly smaller gains in WPD compared with the other three domains (ps all < .006), although again, all four domains saw significant improvements over time (ps all < .001). In terms of effect sizes across the four domains (partial eta squared), Y1’s were SPD (0.521) > WPD (0.309) > Comp (0.215) > Read (0.116) while Y2’s were SPD (0.475) > Comp (0.430) > Read (0.324) > WPD (0.192) so both groups made the greatest gains on speech production. Finally, there were no significant gains or losses from the post-ICAP time point to the 3-month follow-up (ps all > .07), see Figure 1.

#### 3.2.2. CETI: baseline/3-month follow-up

Like Y1, PWA in Y2 made significant gains in their communicative effectiveness (CETI scores), *t*(36) = 2.79, *p* = .008; however, the effect size for this group was smaller compared with Y1. Change scores Y1 = 12.4, Y2 = 7.4, Cohen’s *d* Y1 = 0.91, Y2 = 0.46. MDC_90_ change score (>11.53) was met for 42% of the total cohort.

#### 3.2.3. GAS short-term (baseline/post-ICAP) and medium-term (baseline/3-month follow-up) goals

Y1’s GAS outcomes have recently been published (Doogan et al., 2022) with PWA making significant gains on all four goal categories (short-term, medium-term, long-term and economic). Because Y2 ran for a year in total, we were only able to collect outcomes for short and medium-term (3-month) goals. Y2 PWA made statistically significant gains for both short-term, *t*(43) = 16.31, *p* < .001 and medium-term goals *t*(43) = 9.23, *p* = < .001. The change in short-term goals was considerably larger than Y1 (Y1 = 16 points improvement, Y2 = 23 points) although the standardised effect sizes were similar: Cohen’s *d* Y1 = 2.52, Y2 = 2.50. For medium-term goals the change was larger for Y1 (Y1 = 17 points improvement, Y2 = 11), again the standardised effect sizes were similar: Cohen’s *d* Y1 = 1.58, Y2 = 1.39. In both cases, significantly lower baseline scores for the Y2 group (p < .001) drove the difference in GAS change scores.

### 3.3. Patient Reported Outcome Measures: Y1 and Y2

#### 3.3.1. Quality of life (SAQoL): baseline/3-month follow-up

**Figure 2:**
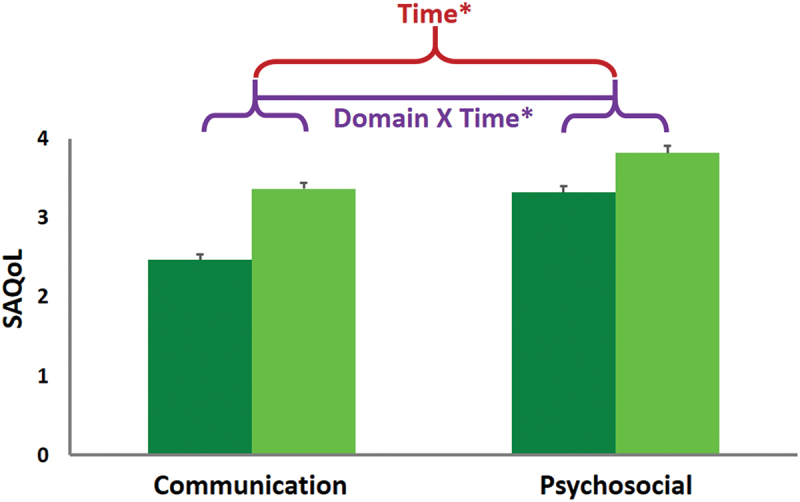
Mean SAQoL outcome scores for the two quality of life domains (communication and psychosocial) at Baseline (dark green) and 3-month follow-up (light green). There was a significant Domain-by-Time interaction with Communication scores increasing significantly more than Psychosocial scores, as well as a main effect of Time (see section 3.3.1 for details). * = P<0.001.

There was no significant Time*Group interaction (*p* = .907), but there was a significant Time*Domain interaction *F*(1, 81) = 21.07, *p* = <.001. This was driven by a main effect of both Time *F*(1, 81) = 105.44, *p* = <.001 and Domain with the Communication component improving 80% more than the Psychosocial component, *t*(82) = 4.62, *p* < .001. Both domains significantly improved from baseline (*p* = <.001), but the effect size was larger for Communication (+0.9, Cohen’s *d* = 1.3) than for Psychosocial (+0.5, Cohen’s *d* = 0.6), see Figure 2. MDC_90_ change score (>0.42) was met for 80% of the total cohort.

#### 3.3.2. Mood (SADQ): baseline/3-month follow-up

There was no significant Time*Group interaction (*p* = .315), but there was a significant main effect of Time *F*(1, 60) = 5.19, *p* = .026, with scores improving by an average of 2.2 points, a medium effect size (eta squared = 0.08).

#### 3.3.3. Confidence (CCRSA): baseline/post-ICAP/3-month follow-up Y2 plus Y1

There was a significant Time*Group interaction *F*(2, 120.8) = 4.05, *p* = .029. This was driven by a main effect of Time *F*(2, 81) = 58.12, *p* = <.001 with Y1 improving more than Y2 between the first two time points, *t*(82) = 2.69, *p* = .009. Y1 improved by 20 points between baseline and 3-months (eta squared 0.58), while Y2 improved by 12 (eta squared 0.27), both large effect sizes.

### 3.4. Carer Reported Outcome Measures: baseline only

Percentage completion rates, central tendencies and variance measures are shown in Table 4. Carers of PWA scored similarly to other diverse carer groups who were involved in the derivation of these CROMs.

**Table 4:** Key baseline variables for carers from Y2. Values are either medians [IQR], ACQOL only, or means (SD). Average scores outside the normal range are shown in the final column.

Variable	% responded	Central tendency	interpretation
ACQOL	89	63 [32]	In line with the mean score: 64
BCOS (10)	46	31.9 (10.8)	Within one SD of the mean score: 35 (6.7)
HADS-D	50	7.1 (5.1)	Within the normal range
HADS-A	50	9.1 (4.7)	Mild anxiety
OCBS-Time	48	44.3 (13.7)	Within one SD of the mean score: 41.5 (11.1)
OCBS-Difficulty	50	32.6 (11.0)	Within one SD of the mean score: 30.3 (12.9)
GSE	50	31.6 (4.3)	Within one SD of the mean score: 32.1 (5.2)

## 4. Perspectives from the therapy team

After the ICAP Y2 finished we asked the team to respond by email in order to ascertain their experience(s) of the ICAP as well those of the participating patients and carers. The reason for this was mainly to inform future iterations of our ICAP. The post-hoc nature of the data collection, without following standard procedures for carrying out prospective qualitative analysis of textual responses, led us to conclude that sharing the raw data is preferable to undertaking a formal qualitative analysis. We asked all team members to independently provide reflections on two aspects of the ICAP: (1) What is your experience of working in a high-dose, high-intensity service compared with standard SLT practice? (2) How do you think the patients and carers experienced the ICAP? The following themes were highlighted by several members of the team: the benefits of working as team and not in silos; how ICAPs offer a unique approach for managing complex profiles of impairment; and, the merits of interprofessional collaboration.

### 4.1. What is your experience of working in a high-dose, high-intensity service compared with standard SLT practice?

AS (Band 5 SLT): “*Working on the ICAP was my first role as a qualified SLT. Now that I have been working in a new role in an inpatient rehabilitation unit, I have been able to better reflect on what made the ICAP so meaningful to the participants and carers. The obvious difference is the intensity and dose of therapy the PWA receive, but also, I believe the timing is important. When people are still in the first few months after their brain injury, they are most often focused on walking and physical recovery, they have also not spent the time adjusting to life in the community after stroke so often do not grasp the impact of their new communication difficulties. Participants in the ICAP have already had the experience to know what they would like to work on as communication goals and have often wanted specific communication therapy for some time but have not been offered this by their local NHS services.”*

LZB (Band 6 SLT): “*I found working in the ICAP more rewarding than standard SLT practice because it was a unique opportunity to focus entirely on patients’ communication needs and deliver doses of therapy that made real changes to the lives of PWA. In over-stretched NHS services, dysphagia work is typically prioritised over aphasia. Working with ICAP participants all day and (nearly) every day for a whole month offered me insights into their capabilities, challenges and motivations, which are impossible to gain through brief or less frequent therapy encounters. These nuanced insights led to more personalised therapy. The comprehensive nature of the programme meant we could work with patients using a wider variety of approaches and formats; I believe this helped each individual find what worked best for them and achieve their true potential.”*

BM (Band 7 SLT): “*As experiences go, the two could not be more different. As speech and language therapists we have become used to assessing, analysing results and treating within a very short time frame, seeing the patient on average once per week for perhaps six weeks if the service allows. Of course, there are exceptions to this. The opportunity afforded to both the patient and myself within the context of high intensity, high dosage therapy allowed me something extra that I hadn’t expected; a chance to get to know the patient. It also granted me the time and space to work with what is important to them. I recall a particularly memorable example of this when a patient, towards the end of the four week programme, gave a presentation on how to make a cake. In planning the session, he had posited the idea of baking the cake and bringing it in for the group to sample, and so he did. Before his stroke, he was a teacher of music and his teaching persona was evident in the session. It became apparent that he was also a very good cook. The culmination of therapy during the previous weeks and the chance to express this through something meaningful was as rewarding to me as it was to the patient.”*

JB (Band 8 SLT): *“ICAP delivery permitted consideration and treatment of all aspects of a PWA’s communication difficulty simultaneously. Managing complex profiles of impairment that are highly individual and require carefully considered modifications to approach and support are far beyond the scope of the standard low dose therapy programmes available via most UK community services. Accordingly, it is not surprising that many community SLT’s favour largely compensatory interventions that can help people with aphasia meet goals without realising their potential. Working together as a team, thinking deeply about patterns of impairment, obstacles to change and possible solutions is hugely rewarding whereas working in isolation within a generalist caseload without the time to work effectively is not.”*

CD (Band 8b Clin Psy): “*The approach we as a team took was a systemic and holistic one which was impressive given that it was what could be described as aphasia boot camp. We all got to know the PWA and their families really well and very quickly. This was striking as the intensity for the PWA was somehow mirrored for the team. This meant we formulated people very early (sometimes before admission) and updated this on at least a weekly basis. After only a couple of days, we had a rich understanding of the complexities and motivations of the PWA and their families. Through the carers assessment and the carers café we also were able to gain collateral on their perspective of the PWA and the system around them. This led to a greater understanding of the support needed for all and I think this added value to the experience and outcomes for the PWA. If there were psychological aspects that needed an individual approach then I could do joint sessions with the key worker and I only wish I had more time to do more of this work.”*

SG (Band 8b SLT): *“The line manager’s role in the UCLH ICAP was to recruit staff, support staff in following local policies and procedures (including leave and sickness), provide supervision as needed, and support integration with the wider SLT team. There were multiple benefits of hosting an ICAP at UCLH. At a profession level, it was fantastic to start addressing the evidence-practice gap for people with aphasia. At a service level, it was extremely positive to foster expertise in working with aphasia and to identify ways to distribute that knowledge across the wider team. At an individual level, it was a privilege to observe the relief in people with aphasia and their carers when they were accepted on to ICAP and to then witness the progress they made in impairment, quality of life, and confidence. However, challenges were experienced. It was logistically challenging to ensure five staff members started at the same time. Given ICAP delivers 6.5 hours of intervention per day, it could be difficult to ensure there was time in the teams’ job plans for integration, CPD, and projects with the wider SLT team; this required calculation of a roster of allocated work to release team members on a rota basis for team activities. Given ICAP worked in a satellite site at UCLH, ensuring the team had easy access to line management required careful consideration. Clear and effective procedures and channels of communication were needed to plan regular annual leave, support with any sickness, and deal with any urgent clinical incidents.”*

APL (consultant neurologist): “*What struck me was the number of PWA and their families who had got stuck in a rut. Some in terms of their therapy, but most in terms of their patterns of communication. An example of the former was one couple who assiduously practiced the same materials that they had been given years ago by their community therapist. These may have been suitable at the time of discharge, but five years down the line, they were not effective. In the latter category were PWA whose communication was effectively outsourced to those around them. We spent quite a lot of time in the Meet the Drs sessions asking carers not to talk, to give space for the PWA to express themselves, even if this took time, they made mistakes or it was socially uncomfortable. Some of the improvements in speech production and communicative confidence came from making sure PWA were given time and space to practice their speech in real world scenarios without anyone stepping in for them.”*

JC (consultant SLT): “*With frequent therapy sessions, therapists could closely monitor clients’ progress and make adjustments to the treatment plan as needed. This allowed for a more dynamic and responsive approach to therapy. Therapists in our ICAP collaborated closely and often conducted joint sessions with fellow SLTs and the clinical psychologist to address the holistic needs of PWA and their families. This interdisciplinary collaboration significantly enhanced the quality of care provided. In routine community care, therapists often work in isolation with a varied caseload- with individuals at different stages of recovery and with various communication disorders. They face limitations in terms of time, funding, and availability of services and equipment of their broad caseloads. This can impact the frequency and duration of therapy sessions and the extent of support they can provide to PWA. Therapists in the ICAP did have more dedicated resources but faced a heavier clinical workload due to the increased frequency and duration of face-to-face therapy sessions. This required careful scheduling and time management to provide effective therapy to multiple clients from multiple therapists.”*

### 4.2. How do you think the patients and carers experienced the ICAP?

LZB: *“At the start of each ICAP patients and carers were clearly delighted to have a place on the programme. Many felt the amount of SLT received previously had been inadequate and were eager to engage in more. By the end, most participants told us they wanted the ICAP to continue for longer. I was surprised by the fact that fatigue (or indeed boredom!) was hardly ever an issue, given the long and intense days. Many participants had little experience of group therapy or meeting others experiencing similar difficulties; the ICAP was transformative in offering them a space where they could feel less alone and receive psychological support. I was particularly struck by the noticeable changes in communication confidence over relatively short periods of time.”*

BM: “*I was fortunate enough to interview a selection of carers and ask them how they think the ICAP has impacted the patients under their care. The most common theme that appears to have emerged is how the patients’ confidence has grown and the impact that has subsequently had on all their lives (see end of the article for some PWA post-ICAP quotes).”*

JB: “*The ICAP was conducted entirely as a face to face programme (rather than a remote intervention) and the benefits to relationship development, unprompted/naturalistic interaction and shared experience were unmistakable. Participants enjoyed themselves and many socialised at break times and in the evenings (i.e., those staying in hotels nearby). Clinicians didn’t count or code separate interactions/types of interaction but the richness and variety of the experience was likely to have been a major factor in achieving the overwhelmingly positive communication confidence (CCRSA) and quality of life (SAQoL) outcomes recorded at 3 months post-treatment. Communication change thrives in high energy, high momentum and high demand situations and whilst participants commonly reported feeling tired, nobody with pre-attendance concerns around fatigue encountered any difficulty. When asked to propose changes to the ICAP model participants’ responses were universally the same: they wanted more and asked for either a six-week programme or the opportunity to come back and do it again!”*

CD: *“Through Acceptance and Commitment therapeutic (ACT) groups, the PWA experienced and explored feelings of being ignored, marginalised and trapped by a lack of control over their altered futures. Some had lived in this isolated world for a very long time They were able to identify ways they wanted others to facilitate their communication and also their own self-imposed barriers namely a reduced confidence in socialising in wider systems. They all named people or groups that they wanted to connect with after the ICAP and knew this would be difficult but meaningful. Working with the carers revealed months or years of, ironically, feeling isolated and silenced. Pre-stroke friendships fell away, and they felt that no one really understood aphasia or its devastating impact. They believed that they should be able to cope and questioned their own resilience. Through sharing their stories and using ACT, many learned to understand the impact and be more compassionate towards themselves. They felt less alone and many kept in touch outside of the programme continuing to offer each other peer support.”*

JC: “*Patients and their caregivers noticed improvements in their loved one’s speech, comprehension, and overall communication skills within a relatively short period. This was highly rewarding and encouraging for both the individual with aphasia and their caregivers – especially when many were years after their stroke. This helped restore ‘hope’. The structured therapy program, ongoing feedback, and noticeable progress kept individuals engaged and motivated to actively participate in therapy activities. This motivation may contribute to better outcomes and a sense of empowerment for patients. Having a specialised team using a collaborative approach helped individuals cope with the challenges of aphasia and provide a sense of reassurance. PWA did report this intensive approach challenging but rewarding. The concentrated nature of ICAPs aims to accelerate progress and facilitate noticeable improvements in communication abilities. PWA, their families (and therapists) reported a sense of achievement and motivation as they witness tangible advancements within a shorter timeframe. This boosted their confidence and overall satisfaction with the therapy program. The intensive therapy schedule also demanded increased dedication and participation in therapy activities. This helped patients to stay focused on their therapy goals and maintain a sense of momentum throughout the program. The increased therapy demands and time commitment was physically and emotionally taxing for some. The availability of resources, including financial constraints also impacted the feasibility and accessibility of ICAP – not all PWA could afford to come (transport, childcare costs, taking time off work).”*

## 5. Discussion

In terms of impairment, the standout result from both years of the Queen Square ICAP is that, on average, large gains were made across PWA’s four main language domains, with speech production improving the most; presumably because talking better features on most PWA’s list of goals. This is in keeping with the majority of published ICAPs (Edna M. Babbitt et al., 2015; Hoover et al., 2017; Leff et al., 2021; Nicholas et al., 2022; Persad et al., 2013). Only one ICAP (LIFT) has failed to show a significant improvement in impairment-based outcomes, this was the first ICAP to report its findings and was likely under-dosed at only two weeks long and with 40 hours of total therapy time (Rodriguez et al., 2013). Later iterations of the LIFT model with higher doses have demonstrated clinical efficacy using both high and low intensity scheduling (J. Dignam et al., 2015). No interventionist wants to improve impairment only; unless associated with comparable gains in communicative function and participation such an effect would represent little more than a party trick. Thankfully, holistic, interdisciplinary and, at times, systemic elements are baked in to most ICAPs ensuring that clinically meaningful gains across the span of the ICF classification are the most common outcome.

Effective ICAPs seem to find a way of providing a high dose of tailored, high quality, multi-dimensional therapy delivered by expert practitioners who enjoy working with their colleagues, PWA and their partners/members of their close social circle. Exactly how one does this seems to matter less; although, having stated this, there were several key structural and PWA characteristic differences between Y1 and Y2 of the Queen Square ICAP that are worth touching on. In response to feedback from the clinical teams, we changed the site, team structure and weekly timetable of the ICAP. The change of site had the advantage of providing more choice of space for the ICAP team to use, although there were times when it was hard to find rooms suitable for group work (size, acoustics). The downside was that in Y2 there was less interaction between the in-patient neuro-rehabilitation treating team and the ICAP treating team. While retaining the same number of staff in the ICAP, we structured the team in a more hierarchical manner with each SLT providing clinical supervision for their less experienced colleague. We also only employed qualified therapists in Y2, thus reducing time needed for delegation. The overall line management was delivered by a senior SLT who was not part of the treating team and provided an independent opinion on how the service was being run. Other than the clinical academics (APL and JC) and one SLT (who was a rehab assistant in Y1 returning as a newly qualified SLT in Y2) the Y2 team were new to the ICAP model of aphasia treatment.

Within the NHS, clinical documentation and other, non-patient facing tasks have risen in recent years to such levels that they negatively impact the time that therapists can spend with their patients (Clarke et al., 2018). We pared this back as much as possible, with the PWA’s weekly timetable as a template for recording which planned activities took place. This also removed the requirement for a narrative handover, as the treating therapist would likely be seeing the patient again later that day or the next. Despite this, feedback from the Y1 team was that there still was not enough time in patient-facing days to manage all the administrative tasks, so, not wishing to compromise total dose, we moved to a four-day a week for four weeks model. The treating staff reported that this model was acceptable, with the only downside being that we able to complete less cohorts in a year i.e., reduced from 16 PWA (4 cohorts) to 12 PWA (3 cohorts) per quarter. Most of the PWA who completed the 4 week ICAP were happy with this model, some said that they would have preferred the three-week model as it meant ‘putting their life on hold’ for less time, while some wanted it to last longer.

In terms of baseline characteristics, Y2 PWA had a longer time since onset than their Y1 compatriots, by almost a year, and they were significantly more impaired (by 21% on average across the four domains of the CAT). Despite this, the ICAP-related changes in previously reported outcomes were generally recapitulated. Regarding effect sizes, both groups’ speech production abilities (as measured on the CAT SPD task) improved the most, likely reflecting the importance that PWA and therapists had put on this in goal setting. In contrast, on the CAT overall, the Y1 cohort improved more on both language production tasks, while the Y2 cohort had superior effect sizes for perception-based outcomes (Figure 1). A possible explanation for this is that Y2 PWA scored particularly poorly on these domains at baseline and this may have prompted therapists to work harder on their language comprehension abilities at the outset. Irrespective, the impairment-based gains observed across both year groups are in line with similarly impressive results from other ICAPs (Rose et al., 2022).

In terms of functional communication effectiveness, groups CETI scores improved following the ICAP, but gains were of a lower size in the Y2 group. Carers score the CETI and it may be that because the Y2 cohort were more severely impaired at baseline, impairment-based gains, especially in language comprehension abilities, may not have been observed to impact daily communication function as much as that observed with the Y1 cohort.

Cognisant of the GAS goal improvements seen in Y1 (Doogan et al., 2022), the therapists took to the idea that they could be more aspirational in their goal setting discussions with the Y2 PWA. This led to more challenging short-term goals being set. The subsequent lower baseline GAS scores for this group drove the relatively greater improvements seen in the Y2 data, rather than any observed over-achievement at the post-ICAP time point. Because we had to terminate the service after a year, we were unable to review longer-term goals at the 6-month time point, which likely caused the smaller gains seen for the medium-term goals (the Y1 published analysis of medium-term goals included a mixture of three and six months scores post-ICAP).

In terms of impact of the ICAP on PWA’s quality of life, participants made clinically and statistically significant gains on our main outcome measure the SAQoL. We recorded data on two of the three domains (Communication and Psychosocial) and both improved by more than double the responsiveness to change previously published for each (Hilari et al., 2009), with an interaction (Communication > Psychosocial) likely reflecting that SLT sessions outnumbered psychological sessions ~ 10:1. Although, on average at a group level PWA who took part in our ICAP did not score in the depressed range at baseline on the SADQ, participation in the programme made a very big difference to their self-confidence ratings and certainly seemed to have lifted overall mood ratings. Both effects were measured at the 3-month follow-up, highlighting the lasting change that ICAPs can produce.

Chronic aphasia clearly has systemic effects, causing a shrinkage of the social networks and the number of people available to share the burden of caring for PWA (Doogan & Leff, 2021). The baseline carers’ assessment data is broadly in line with other surveys of carers of people with acquired brain injury and highlights another important source of unmet need. We were disappointed that we were unable to collect enough data to meaningfully examine the effects of the ICAP on carers. We think that they are a vital part of the support system of the PWA and need to be assessed more fully in future iterations of ICAPs. Recently set-up ICAPs have taken this on board and offer more therapy directed at carers or family members than previously (Rose et al., 2022). While other ICAPs are starting to include therapists trained in psychological interventions, we think ours is the first to offer a Carers’ Café that provides psychologist-delivered group support, with the aim of these carer cohorts becoming self-supporting. At least two of our carer groups continue to meet.

The teams’ reflections largely speak for themselves. Most express how valued and valuable they have found being part of this iteration of an ICAP. Staff burnout was an issue in the Y1 model; many of the changes in Y2 were in response to this. The treating team valued the quarterly reflection weeks and these were used to openly discuss all areas of our service and to explore and implement changes to the ICAP model, a practice that should perhaps be adopted by other teams delivering complex interventions. Harking back to the Ericsson quote at the top of this paper, it is perhaps no surprise that the majority of participants (both PWA and their carers) have noticed meaningful changes in their language abilities and manner of communication. However, seeing these gains in real time is rewarding and speaks to the therapeutic power of what a team of expert human coaches can achieve with groups of people with acquired brain injury. If a drug caused this effect over a range of communicative and affective outcomes, it would be all over the world’s press.

It is perhaps surprising that no RCTs have been carried out on the ICAP model. Given the chronic nature of the condition that sees most PWA attend ICAPs years into their stroke, practitioners in the field seem convinced that the impressive effects that we see are genuine and are not driven by chance, bias, confounding or the effects of time. However, those who hold the purse strings of health care budgets do not appear to be as impressed as we are by the steady stream of independent, confirmatory evidence. The funding for our ICAP has come to an end. The team has disbanded and with a post-COVID hangover affecting NHS commissioning of new clinical services within the UK, it is not clear if or when we can restart version three. Experts do not come cheap (and nor should they). A clear challenge for our community is to provide evidence that ICAPs are economically as well as clinically effective. It may be that next steps include clinical trials of the ICAP model with economic outcomes clearly imbedded. The holistic and systemic impacts of ICAPs are becoming clear, but their wide-ranging effects cause a problem when it comes to fiscal measurement. How best to capture this across multiple areas of governmental budgeting (primary and secondary health services, mental health services, social services, taxable income from getting PWA and carers back into paid work, savings on state benefits) remains unresolved. Certainly, the oft-cited EQ5D is not a solution, as it does not capture changes in either cognition or communication.

It seems fitting to finish this paper with some quotes from PWA’s and carers exit interviews, which may help inspire other health care professionals to pursue the ICAP model:

“First week scared then fantastic!”

“Thursday reading and it’s much better … a lot better … and all the time yesterday talking a lot.”

“It’s given me confidence. I have to go and use that confidence in my daily life”

“The Carers’ Cafe has been an unexpected gift. To share and liaise with other partners of stroke survivors who have Aphasia has been a rare opportunity. To come together after the worst stages of shock has been very special. Our experiences have similarities and differences and we have all experienced isolation. This forum with professional support has given me a chance to reflect on my needs as a carer rather than just my partner’s and at a time when I am less overwhelmed by tears. It has enabled me to plan and work towards goals which enhance my day to day experience and consequently nourish us both. I would recommend this service to all in my situation if it was available.”

“_____ has been brilliant at practicing speech and everyone has noticed a difference. He’s doing a running commentary when in the kitchen. He came out for a meal and ordered himself, we went to RHS garden yesterday and he asked for tickets, and ordered lunch, we’ve got a FaceTime booked tomorrow night with his friend in Spain and a couple of others have said they will do this to. He’ll be walking to _____’s session each week and checking in (he did that well last week with me). Finally, he has said he will go to the local carpet bowls twice a week at the village hall, which is amazing! This wouldn’t have happened without you, so thank you so much.”

“AMAZING NEWS! Mum went into _____Nursery today by herself and spoke to the headteacher. She offered her a volunteer role … They are just sorting out a DBS check for her in the meantime. Thanks again for helping!”

“Brain cells I haven’t used for a long time”

“Big words”

## Supplementary Material

Supplemental Material

## Abbreviations

ACQOL: Adult Carers Quality of Life questionnaire
BCOS: Bakas Caregiving Outcomes Scale
CAT: Comprehensive Aphasia Test
CCSRA: Communication Confidence Rating Scale for Aphasia
CETI: Communicative Effectiveness Index
CROMs: Carer Reported Outcome Measure
GAS: Goal Attainment Scale
GSE: General Self-Efficacy Scale
HADS: Hospital Anxiety and Depression Scale
ICAP: Intensive Comprehensive Aphasia Program
OCBS: Oberst Caregiving Burden Scale
PROMs: Patient Reported Outcome Measure
PWA: People with aphasia
SADQ: Stroke Aphasic Depression Questionnaire
SAQoL: Stroke and Aphasia Quality of Life Scale
SPD: Spoken Picture Description
WPD: Written Picture Description
