# Supplementary material for: More than one way to improve a CAT: Outcomes and reflections on two iterations of the Queen Square Intensive Comprehensive Aphasia Programme
Source: Aphasiology. 2023 Dec 6;39(1):40–63. doi: 10.1080/02687038.2023.2286703 (PMC11027995; doi:10.1080/02687038.2023.2286703)
Supplement: Supplemental Material [file PAPH_A_2286703_SM2963.pdf]

Intensive Comprehensive Aphasia Programme @ Queen Square

Operating Manual

Page 1. Title and contents
Page 2. Purpose
Page 3. Objectives
Philosophy and model of care
Page 4. Estates, contact methodologies and mode of operation
Page 6. Staffing – roles and responsibilities
Page 10. Service information
Page 12. ICAP pathway
Page 13. Referral management
Service Provision
Page 13. Keyworker role
Assessment
Page 17. Documentation
Page 19. EXAMPLE Clinical timetable
Page 26. Regular GROUP therapeutic activities
Page 36. Regular INDIVIDUAL/PAIRED therapeutic activities
Page 46. GOAL SETTING, review and scoring
Page 48. Discharge reporting
Page 49. APPENDICES

A. Discharge report (example)
B. Queen Square ICAP Equipment Inventory
C. Goal setting support tool
D. MY APHASIA and how to support me presentation slides
E. 2022 CLINICAL REFLECTIONS (recommended changes to ICAP)

Purpose

Aphasia is an acquired language disorder most commonly resulting from stroke. Approximately 31% of all first-time strokes lead to some degree of aphasia and 60% of people affected will continue to present with the condition beyond 12 months – the overwhelming majority for the rest of their lives. There are approximately 350,000 people living with aphasia in the UK currently, but this is expected to rise substantially in the next decade. King et al. (2020) have projected that the incidence of stroke in the UK will increase by 60% between 2015 and 2035 accompanied by significantly reduced rates of mortality.

The difficulties understanding and producing language (speech and text) that are the defining characteristic of aphasia often lead to profoundly negative and long-lasting psychosocial consequences. Most people with chronic aphasia are unable to work, experience altered relationships and familial role, become dependent on others for everyday tasks that involve language, frequently become withdrawn and often experience high levels of frustration. The incidence of depression after aphasia is estimated to be 62 % to 70 % (higher than in stroke survivors who do not have aphasia). The burden of care for families of people with aphasia is substantial and individuals who support people with aphasia are also prone to developing depression. People with aphasia report difficulty accessing existing mental health services because of their communication disability and this is also true for non-specialised intervention programs or support groups that could theoretically meet some of their needs.

Systematic reviews of aphasia therapy support the hypothesis that the greater the number of therapy hours, the larger the gains in language recovery in the chronic stage post-stroke. People with aphasia benefit most if they have an average of more than 100 hours of therapy but this optimal, evidence-based recommendation is considered out-of-reach for most statutory (NHS provided) therapy services in the UK where a person with aphasia might reasonably expect ≈8 sessions in total*.

An Intensive Comprehensive Aphasia Program (ICAP) offers an efficient means of delivering the high dose, high intensity therapy that research recommends for clinically meaningful gains in language performance and meaningful changes to functional communication. By definition ICAPs must provide high-intensity therapy at a minimum of 3 hours per day over at least 2 weeks using a range of formats and treatment approaches (including group and individual therapy) to target impairment, activity and participation levels of language and communication functioning in line with the World Health Organisation International Classification of Functioning framework. They must also supply aphasia education/recovery promotion advice for the individual and family, as well as having a defined start and end date with a set cohort of individuals entering and leaving the program together. The Queen Square ICAP embodies all of these elements and also incorporates a neuropsychological

component to support the holistic and systemic recovery of people with aphasia and their families.

**In 2015-2016, people with aphasia received an average 3.6 hours of Speech and Language Therapy as an inpatient and a further 6.3 hours in the community (Fulop et al. Health Services Delivery Research 2019;7(7), Palmer et al. PloS one. 2018;13(7)). Evidence indicates an average of 98.4 hours of Speech and Language therapy (SLT) is required to achieve positive functional communication outcomes (Bhogal et al. Stroke. 2003; 34:987).*

Objectives

The Queen Square ICAP aims to deliver expert, high-intensity, evidence-based rehabilitation within an interdisciplinary model of working. People with aphasia can be referred by their GP from anywhere in the UK and suitability is determined via an initial assessment consultation. The ICAP operates as an outpatient service with participants attending daily four consecutive days a week for four weeks. Clearly defined goals are devised for attainment during the period of attendance and medium to long-term goals are set in the final week of the program. Participants receive a comprehensive report following completion of the ICAP and recommendations are made to help promote further recovery. Appropriate referrals are completed to local services, groups and organisations at discharge in an effort to maximise lasting positive change.

Philosophy and model of care

The ICAP is aligned with the UCLH vision and is committed to delivering top-quality patient care, excellent education and world class research. The program is, at the time of writing, the only publicly funded ‘free at the point of need’ intensive aphasia treatment in Europe and it contribute to the evidence base for more widely available intensive therapy provision.

UCLH’s values of SAFETY, KINDNESS, TEAMWORK and IMPROVING are central to the everyday operation of the ICAP.

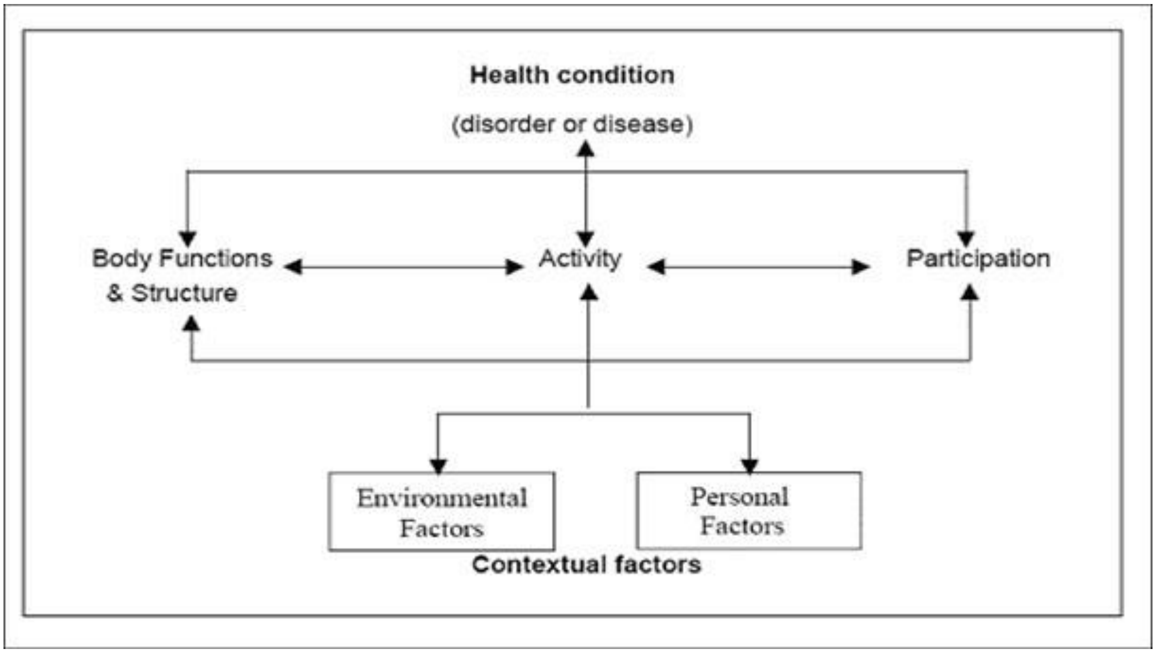

The ICAP model of care adopts a patient-centred goal driven approach which simultaneously addresses all aspects of the World Health Organisation's International Classification of Functioning, Disability and Health (WHO ICF) to generate measurable changes to the person with aphasia's communication skills, participation and psychosocial wellbeing (WHO 2002). Simultaneously addressing all aspects of the WHO-ICF has been found to produce the best outcomes (Rose et al. Topics in Stroke Rehabilitation. 2013;20(5):379).

Comprehensive interdisciplinary intervention is tailored to the person with aphasia's personal goals, profile of aphasia, cognitive ability and the individual's psychosocial needs, resources and priorities (as assessed prior to intervention via formal and informal means including discussions with family and friends).

Personal factors could be considered the internal context from which a person with aphasia approaches activity and participation. Such factors can have a profound impact on perceived quality of life and the rehabilitation outcomes of people with aphasia. The World Health Organisation defines quality of Life as: "An individual's perception of their position in life in the context of the culture and value systems in which they live and in relation to their goals, expectations, standards and concerns". Quality of life is not conceptualised within the ICF. Kagan et al. adapted the ICF adding the concept of quality of life in the Framework for Outcome Measurement (FROM).

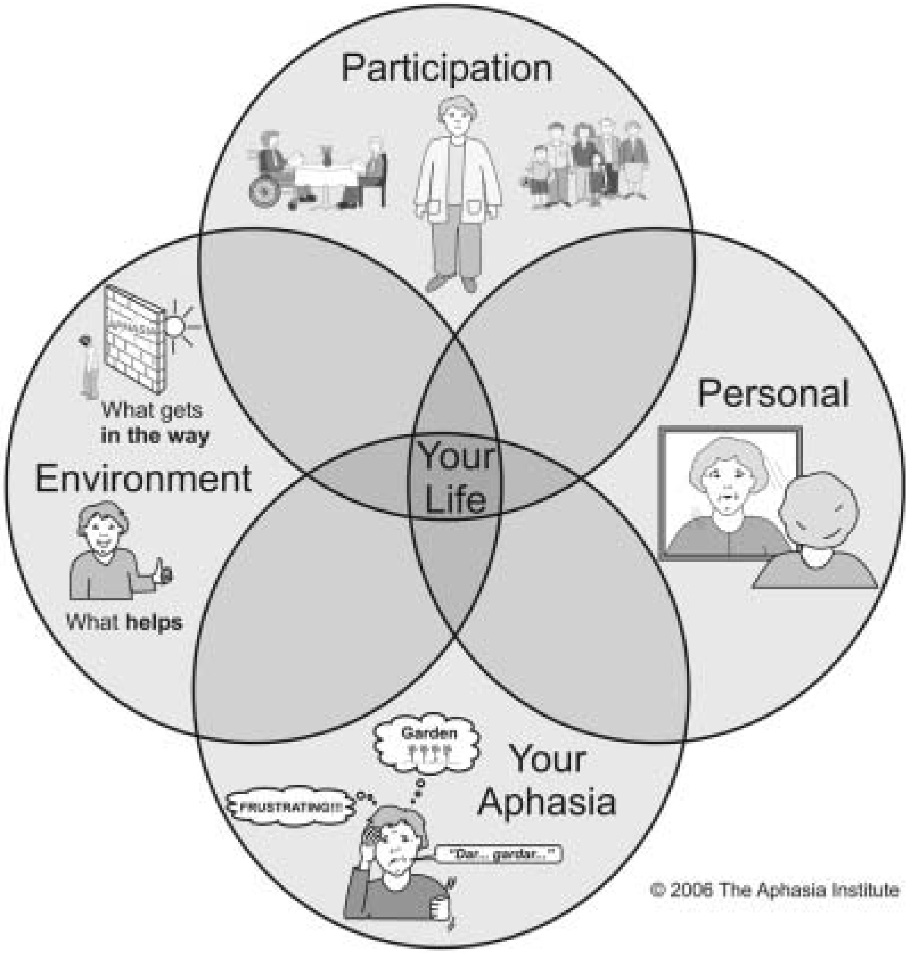

FROM highlights the dynamic and overlapping impact of the ICF domains and how these domains contribute to quality of life. A systematic review identified depression, communication disability, engagement in activities and diminishing social networks as the factors that most affected quality of life for people with aphasia. These factors are widely spread across the domains of the ICF. Given the wide ranging and devastating impact of aphasia on the lives of those living with the condition it is important that treatment addresses all domains of the ICF in order to best impact the quality of life of people with aphasia.

Estates, contact methodologies and mode of operation

The Year 2 (2022) Queen Square ICAP operated from NHNN premises at 25 Cleveland Street (W1T 4AJ) and made use of at least 3 individual clinic rooms each Tuesday through to Friday with a group room. Storage space was extremely limited and all physical resources were situated on wall shelving. Patient related information and assessment data was filed and stored in a lockable cabinet. Valuable equipment such as IPADs and laptops were also stored in the lockable cabinet of this room.

Computer access was achieved via networked machines in each of the allocated clinic rooms and also in the ground floor clinical administration offices via 'hot-desking' arrangement. Networked printers were available.

The 2022 Queen Square ICAP did not have use of any consistent telephone number or internal extension number on site at 25 Cleveland Street and relied on personal mobile phones to communicate within the team and with ICAP service administration. All ICAP participants and their families were issued with contact details for the NHNN Aphasia Service administrator and messages would be relayed to the clinical team via email or personal mobile dependent on urgency. The administrator telephone number was manned between 9am and 5pm weekdays and cover for annual leave/sickness was available from with the wider administrative team. A 'treating team' Whatsapp' group was created for day to day within-team communications and an ICAP URGENT 'Whatsapp' group was maintained to transmit messages to the whole team.

The ICAP operated as an outpatient service 09.00 to 16.15 on clinic days (every Tuesday to Friday). The NHNN premises at 25 Cleveland Street were open and manned from 08.00 to 17.00 by staff affiliated to the UCLH pain management service and it was not uncommon for patients to arrive as early as 8.15 am or leave as late as 5pm when transport arrangements faltered. Occasionally patients arrived as early as 7.50am and were collected as late as 7pm in exceptional circumstances (i.e. impact of train strikes or traffic disruption on taxi availability). The clinical team staggered working hours in an effort to accommodate variance and support patients' needs at these times (2 members of the treating team working between 08.15 and 16.15 and 2 members working between 08.45 and 16.45). Participants attended the ICAP as day patients and the clinical team did not hold a duty of care beyond treatment on-site.

Staffing - roles and responsibilities (brief summary)

ICAP staffing (Jan 2022) is shown in the table below:

**Staff**	**Grade**	**Whole time equivaler**
Neurologist	Consultant	0.1
Speech and Language Therapy	Consultant SLT<br>Band 8a<br>Band 7<br>Band 6<br>Band 5	0.1<br>1.0<br>1.0<br>1.0<br>1.0

Clinical Psychology	Band 8b	0.5
Administrative support	Band 4	0.5

Consultant neurologist:

- Provides medical oversight and operates a single 'meet the doctors' opportunity for one hour for each ICAP cohort – fielding questions about medical concerns and stroke aftercare (initiates onward referrals).
- Weekly attendance of ICAP ward round – contributes to interdisciplinary planning/ decision making related to ICAP participants and holds a senior level of responsibility for service management and operational decisions.
- Responsible for leading data analysis and research reporting.

Consultant Speech and Language Therapist:

- Operates the APLJC Aphasia Clinic on a twice monthly basis (1st and 3rd Friday of each month) via honorary contract from UCL with support from the Aphasia Service administrator. Role involves clinical assessment/review and provision of advice to people with aphasia referred for ICAP consideration (with determination of each candidate's suitability for the Queen Square ICAP). Role also demands varying levels of aftercare with community referrals, signposting to available group and support resources and preparation of a comprehensive report (to attendee and referring GP) all typical.
- Weekly attendance of ICAP ward round – contribution to interdisciplinary planning/ decision making related to ICAP participants. Also holds a senior level of responsibility for service management and operational decisions.
- Involved in data analysis and leading research reporting.

Clinical lead – Band 8a Speech and Language Therapist:

- Jointly involved with strategic vision of the service
- Responsible for day to day operation of the ICAP.
- Management and supervision of the clinical team (aided by NHNN clinical manager re. administrative aspects of line management – booking leave/sickness reporting etc.)
- Responsible for quality of assessment, intervention and post-treatment recommendations with a focus on ensuring equity of experience for all ICAP participants
- Keyworker responsibility for one ICAP participant and deputy keyworker responsibility for one other participant per cohort
- To maintain timely documentation – upload to EPIC and regularly input all assessment data into outcome information recordings
- Responsible for maintaining a complete and accurate dataset of outcome

information.
- Leadership in the education of staff and support with continuing professional development
- Ensures programme compliance with UCLH infection prevention, information governance and risk policies.
- Responsible for liaison with external stakeholders (i.e., clinicians from other trusts) and development of professional relationships with related agencies (i.e., non-statutory aphasia services).
- Preparation and delivery of service-related content (conference presentations /engagement with media etc.)
- Organisation and attendance of ICAP ward round with 'chair' responsibilities – contribution to interdisciplinary planning/ decision making related to ICAP participants.
- Close liaison with aphasia service administrator
- Clinical supervision for band 7 SLT

Band 7 Speech and Language Therapist:
- Supports day to day operation of the ICAP.
- Supports the ongoing education of staff - continuing professional development
- Contributes to the development and provision of quality assessment, intervention and post-treatment recommendations with a focus on ensuring equity of experience for all ICAP participants
- Keyworker responsibility for one ICAP participant and deputy keyworker responsibility for one other participant per cohort
- To maintain timely documentation – upload to EPIC and regularly input all assessment data into outcome information recordings
- Supports clinical lead with integrity of outcome information recordings
- Supports programme compliance with UCLH infection prevention, information governance and risk policies.
- Preparation and delivery of service-related content (conference presentations) in support of clinical lead
- Weekly attendance of ICAP ward round with 'deputy chair' responsibilities – contribution to interdisciplinary planning/ decision making related to ICAP participants.
- Close liaison with aphasia service administrator
- Clinical supervision for band 6 SLT

Band 6 Speech and Language Therapist:
- Contributes to day to day operation of the ICAP.
- Contributes to the ongoing education of staff - continuing professional development

- Contributes to the development and provision of quality assessment, intervention and post-treatment recommendations with a focus on ensuring equity of experience for all ICAP participants
- Keyworker responsibility for one ICAP participant and deputy keyworker responsibility for one other participant per cohort
- To maintain timely documentation – upload to EPIC and regularly input all assessment data into outcome information recordings
- Supports programme compliance with UCLH infection prevention, information governance and risk policies
- Preparation and delivery of service-related content (conference presentations) as appropriate
- Weekly attendance of ICAP ward round – contribution to interdisciplinary planning/ decision making related to ICAP participants
- Close liaison with aphasia service administrator
- Clinical supervision for band 6 SLT

Band 5 Speech and Language Therapist:

- Contributes to day to day operation of the ICAP.
- Contributes to the ongoing education of staff - continuing professional development
- Contributes to the development and provision of quality assessment, intervention and post-treatment recommendations with a focus on ensuring equity of experience for all ICAP participants
- Keyworker responsibility for one ICAP participant and deputy keyworker responsibility for one other participant per cohort
- To maintain timely documentation – upload to EPIC and regularly input all assessment data into outcome information recordings
- Supports programme compliance with UCLH infection prevention, information governance and risk policies
- Preparation and delivery of service-related content (conference presentations) as appropriate
- Weekly attendance of ICAP ward round – contribution to interdisciplinary planning/ decision making related to ICAP participants
- Close liaison with aphasia service administrator

Band 8a Clinical Psychologist:

- Conducts initial ‘systemic’ psychological assessment of carers ahead of ICAP attendance (usually at the same time as initial baseline assessment)
- Facilitates weekly ADJUSTMENT GROUP with the support of an SLT
- Facilitates weekly CARERS’ CAFÉ
- To maintain timely documentation – upload to EPIC and regularly input all pre-post

assessment data into outcome information recordings
- Supports programme compliance with UCLH infection prevention, information governance and risk policies etc.
- Preparation and delivery of service-related content (conference presentations) as appropriate
- Weekly attendance of ICAP ward round – contribution to interdisciplinary planning/ decision making related to ICAP participants

Band 4 Administrator:

- Contacting matched ICAP candidates from the waiting list and assembling cohorts from those matched candidates that are able to attend given dates.
- Organisation of all hotel bookings and taxi bookings for ICAP candidates (including maintenance of spreadsheets re. booking codes/costings/invoice details etc.)
- Scheduling all baseline and post-ICAP review assessment appointments, confirming with clinical team and updating outlook calendar – responsible for booking any additional transport where appropriate
- Preparing individual participant folders ahead of attendance (NAMED FOLDER with recording document and discharge report template incorporated)
- Saving all ICAP discharge reports to EPIC (when notified by the clinical team) and dispatching copies to all specified recipients
- Printing and posting other ICAP correspondence (upon clinician request)
- Attending ward rounds on a reduced basis (i.e. once per month – ahead of new cohort intake). Having liaised closely with families ahead of attendance the service administrator could often provide important information and insights not yet available to the team.

Service information

Contact details for the aphasia service:

The National Hospital for Neurology & Neurosurgery
Queen Square, London WC1N 3BG

Tel: 0203 448 3848

Email: uclh.nhnn.aphasia@nhs.net

ICAP pathway

GP referral

Suitability assessment

Good ICAP candidacy | Limited ICAP candidacy

Report, liaison with local community SLT, onward referral & recommendations

Waiting list for ICAP | Review (x1 session) and discharge

Pre-ICAP baseline assessment & systemic interview (with principal carer)

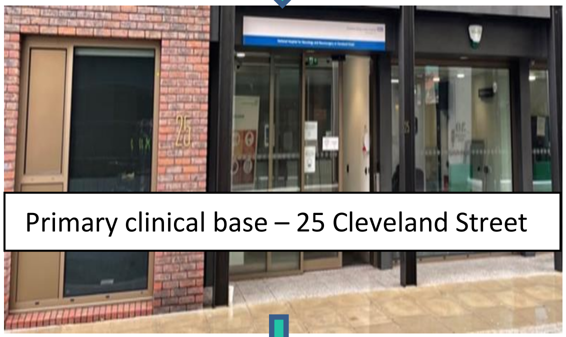

Primary clinical base – 25 Cleveland Street

4 participants 4 week programme 4 days a week (6.5+ hours per day) = 100 hours of therapy

Keyworker assignment & short-term goal setting (using Goal Attainment Scaling GAS)

- Individual therapy
- Paired therapy
- Group therapy
- Community based therapeutic activities
- Family/systemic education
- Weekly ‘adjustment group’ + carer education & support group ‘carer’s café’

Post-attendance assessment & medium term/long term/economic goal setting (GAS)

Discharge reports + recommendations + onward referrals

4-6 sessions of remote ‘carer’s café’ support

12 weeks post-attendance review assessment

Discharge

Referral Management

UK citizens over 18 years of age with a diagnosis of aphasia acquired following some form of brain injury (i.e., stroke) are able to be referred to this service.

Good candidacy for ICAP is determined by ascertaining the following:

- Some insight into communication deficits and corresponding motivation
- Some meaningful spoken output
- Some ability to repeat spoken words (at single syllable level i.e. CAT)
- Absence of moderate or severe apraxia of speech
- Evidence of communicative intent and motivation to improve
- Ability to tolerate sustained intervention (for up to 7 hours daily)
- Ability to attend the programme for 16 days with any support needs to be provided by family or friends.

Service Provision

Administrative support

See Band 4 administrator - role and responsibilities (page 7)

Non-clinical day

The year 2 Queen Square ICAP model allocated 4 days of the week to direct clinical treatment and preserved a single working day for interdisciplinary discussion, planning, resource development, pre-attendance assessment and general administration duties.

Whole team meeting

A weekly “ward round” involving all members of team (excepting administrator – attending on a monthly basis as previously specified) was scheduled to take place in the morning of the non-clinical day and permitted the following:

- Planning structure and scheduling of the programme (including making necessary revisions to timetable or process)
- Familiarisation with forthcoming cohort participants – sharing assessment data and using psychological formulation approach to develop a shared understanding of the individual’s system, named objectives and potential obstacles to change
- Review of current ICAP participants’ progress (in turn) with reference to therapeutic priorities and goal achievement. Broad clinical discussion and problem solving related to hypotheses, interventions, challenges, medical

concerns/need for onward referral and family engagement/need for additional education and/or support

- Other business – equipment/ research projects/forthcoming presentations/engagement with stakeholders etc.

Keyworker role

Each participant is allocated a keyworker on admission to the service. The keyworker acts as the primary point of contact for the participant and their family and should complete the individual's initial 'baseline' assessment where possible

Duties as follows:

- Support/complete participant 'systemic' formulation (with psychology)
- Responsibility for key client's daily documentation – ensure that all treating team clinicians complete documentation for all sessions
- Support key client to identify short term goals (day 2) and generate a SMART goal for each stated goal
- Upload all short term goals, SMART wording and initial scoring to the GAS data set within the key client's recording document
- Each full-time therapist should also assume a deputy role for a specified client other than their primary key client. Familiarity with family, goals and therapeutic priorities should be maintained in order to support the primary keyworker and provide effective cover without any discernible loss of focus during episodes of staff leave.
- Support key client to identify medium term & long term/economic goals (day 15 or 16) and generate a SMART goal for each stated goal – upload these to the relevant recording component of the client's recording document and apply all weightings and baseline scores.
- Conduct review assessment (day 15 or 16) <u>and</u> 3 month review assessment where possible.
- Complete key client's discharge report, initiate any onward referrals and liaise with relevant parties as appropriate.
- Upload all assessment and goal scores to the master outcomes recording dataset.

<u>Assessment</u>

Pre-admission baseline assessment (Week 0)

Scheduled for 11am Monday and 1pm Thursday every second and third week of the programme (4x assessments in total per 4x week cycle)

3 hours allocated for baseline assessment and scoring:

- Appointments are scheduled under code JC01 in EPIC

- Attendance is usually agreed, confirmed and scheduled (with any special transport requirements organised) by service administrator
- ‘Check in’ patient on EPIC electronic record system
- Meet the patient, introduce the service and explain the baseline session
- Ask if participant is consenting to recordings (video/audio/both for all purposes/teaching purposes/other) and complete relevant recording forms (form… or pictorially supported form….) Ensure form is signed.
- Use one of the ICAP ipads to record (if participant is consenting) spoken responses to the following questions:
  - What are you expecting from the ICAP?
  - What do you hoping to change?
  - What do you hope will change for your family?

    *Recordings require systematic upload to patient folders within the appropriate shared drive
- Carry out all baseline assessments with the participant: CAT (in its entirety excepting the disability section), SAQuOL 39 and CCRSA
- During administration of the Comprehensive Aphasia Test (CAT) adhere to discontinue rules (4 consecutive errors for spoken sentence comprehension and written sentence comprehension, 8 consecutive errors for object naming only)
- When assessing Spoken picture description only issue prompts if participants do not attend to any of the key areas and ensure that no more than 2 Prompts are provided. Record the nature of each prompt (i.e. <indicated alternative area of picture> OR <“anything else happening?”>). Score speed relative to the quantity of information conveyed in a minute (>75 words in 1 minute = 3, >50 words in 1 minute = 2, >25 words in 1 minute = 1 are suggested). NB. These guidelines do not feature in the CAT manual but are considered valuable when assessment and scoring is being conducted by several clinicians. Specified guidelines help to ensure consistency across raters.
- When supporting participant self rating on the SAQuOL and/or CCSRA clinician’s may reword statements and provide additional support to ensure that a participant has understood correctly.
- Carers should be asked to supply all completed carer-rated questionnaires (CETI, SADQ, AQUOL) and a note should be taken of the person who completed the scoring (to ensure consistency across time-points)
- Carers should be supported to complete the CETI, SADQ and AQUOL if not already completed
- Remind participants of the importance of goals during the programme (helps to spur forethought and preparation)

  Score assessment findings (using the support tools available at:)

Note that the SADQ and AQUOL employ scoring systems that change order to suit the valance of the question wording – reference to the scoring system is advised)

- Document attendance in SOAP format on EPIC and 'check out'
- Transfer scores for all assessments to the master OUTCOMES RECORDING Excel spreadsheet
- Add information to the formulation document (in the participant's folder)

End of ICAP review assessment:

Typically scheduled so that 2 ICAP participants undertake review assessment on day 15 of the programme (morning slot 10.30-12.15) and 2 ICAP participants are reviewed on day 16 (morning slot 10.30-12.15). This arrangement was found to reduce pressure on staffing (particularly when annual leave was a factor) and was viewed as optimal from a participant performance perspective (avoiding after-lunch fatigue).

2 hours allocated for assessment and additional admin time scheduled for scoring:

- Review short-term goal performance and revise scores in the GAS formula tool (built in to the participant's EXCEL recording document.
- Upload 'final score' and 'change in score' to the master outcomes spreadsheet.
- Repeat the Comprehensive Aphasia Test (CAT) and apply all subtests – even those where a participant may have scored above 'aphasic cut-off' at baseline. The only exception to this rule is perhaps WRITTEN COPYING where a robust score of 27 is very likely to be replicated and can be carried over to review
- Facilitate repeat self-rated assessment with the CCRSA, score (total score from 10 items)
- Discuss medium term goals and set at least 2 objectives for attainment before the scheduled 12 week post-ICAP review.
- Discuss longer-term goals and explore the potential for an economic goal (i.e. any goal that benefits the individual, their partner/family or local community economically…. paid or unpaid/voluntary work, carer returning to work of any kind, increased contribution to local economy – shopping/returning to golf club etc.).
- Upload all CAT, CCRSA and goal scores to the master EXCEL outcome spreadsheet.

3 month post-ICAP review assessment:

Typically scheduled on MONDAYS (non-clinical day) and timed to avoid any 'next cohort' initial baseline assessment that might be scheduled for 11am (usually 1 or 2 pm).
One participant usually seen each week across a 4 week period – dates and timing of 12 week reviews are plotted when a cohort completes and the provisional date/time is

included in the participant's discharge report. They may wish to change this via contact with the ICAP administrator.

2 hours allocated for assessment and additional admin time scheduled for scoring:

- 'Check in' patient on EPIC electronic record system
- Use one of the ICAP ipads to record (if participant is consenting) spoken responses to the following questions:
  - What has changed since the ICAP?
  - Has anything changed for your family?
  - Anything else? (i.e., related to goals)
    *Recordings require systematic upload to patient folders within the appropriate shared drive
- Review medium-term goal performance and revise scores in the GAS formula tool (built in to the participant's EXCEL recording document).
- Review long-term goals and/or any ECONOMIC goal
- Add the SMART description of all medium term and long-term/ECONOMIC goals to the master outcomes spreadsheet
- Upload 'medium term goal' GAS 'final score' and 'change in score' to the master outcomes spreadsheet and detail whether they are achieved/partially achieved or not achieved
- Repeat the Comprehensive Aphasia Test (CAT) and apply all subtests – even those where a participant may have scored above 'aphasic cut-off' at baseline. The only exception to this rule is perhaps WRITTEN COPYING where a robust score of 27 is very likely to be replicated and can be carried over to review. During administration of the Comprehensive Aphasia Test (CAT) adhere to discontinue rules (4 consecutive errors for spoken sentence comprehension and written sentence comprehension, 8 consecutive errors for object naming only)
- When assessing Spoken picture description only issue prompts if participants do not attend to any of the key areas and ensure that no more than 2 Prompts are provided. Record the nature of each prompt (i.e. <indicated alternative area of picture> OR <"anything else happening?">). Score speed relative to the quantity of information conveyed in a minute (>75 words in 1 minute = 3, >50 words in 1 minute = 2, >25 words in 1 minute = 1 are suggested). NB. These guidelines do not feature in the CAT manual but are considered valuable when assessment and scoring is being conducted by several clinicians. Specified guidelines help to ensure consistency across raters.
- When supporting participant self-rating on the SAQuOL and/or CCSRA clinician's may reword statements and provide additional support to ensure that a participant has understood correctly.

- Carers should be asked to supply all completed post-ICAP carer-rated questionnaires (CETI, SADQ, AQUOL) and clinician should check that the same individual ha completed the forms as for the initial baseline.
- Carers should be supported to complete the CETI, SADQ and AQUOL if not already completed
- Document attendance in SOAP format on EPIC and ‘check out’
- Upload all CAT, SAQUOL-39, CCRSA, CETI, AQUOL, SADQ and goal scores to the master EXCEL outcome spreadsheet.
- Produce a letter to the participant acknowledging their attendance for 3 month review (in the EPIC electronic records system) incorporating a summary of their assessment performance and including details of GOAL performance with any further advice
- Discussion regarding operational issues i.e. staffing levels (i.e. managing sickness or leave), equipment needs, issues relating to estates/room bookings etc.
- Planning and review of internal projects including within service audit, preparation of articles for publication, attendance and presentation within appropriate professional forums (i.e. Aphasia symposium etc.)
- Planning and preparation for ICAP reflection week (non-clinical week every 12 weeks – dedicated to administration, reflection and service review as continuing professional development needs)
- Discussion re. management and professional issues such as mandatory training, policy and procedural updates and DATIX reporting
- Planning engagement with associated relevant professionals and media operators to share operational expertise and develop awareness of ICAP model, rationale and outcomes

Documentation

Documentation demands were kept to a minimum given that clinicians are required to deliver direct therapy for >6.5 hours a day and cover leave within the team. All therapy ‘session’ notes were written into each participants’ individual EXCEL recording document (stored in their individual folders) within 24 hours of the session or activity. Where an activity title and description was pre-written the clinician need only write a summary of the outcome (i.e. score or a brief note on performance). Where there was no corresponding activity title or description this must be added to the activity column at the beginning of the document (ending with a colon : and a single space – which helps delineate activities and outcomes in the composite text. Once written the clinician can add a summary of performance and the title/description will auto populate ahead of the outcome (performance

summary) in the cumulative activity box for the day (located below the activity grid with columns for each day of the 16

Short term GAS recording & formula

BANK of regular group, 1:1 and paired therapy activities: COPY & PASTE to descriptor column

MTG and LTG GAS recording

16x daily recording columns related to activity descriptors (supported by formula) TYPE HERE

All daily documentation auto-populates preceded by activity description
SAVE FROM HERE
(DO NOT TYPE)

IGNORE documentation carries to this field to support concatenation

MASTER recording document is located at:

M:\ICAP Intensive Comprehensive Aphasia Program\ICAP YEAR 2

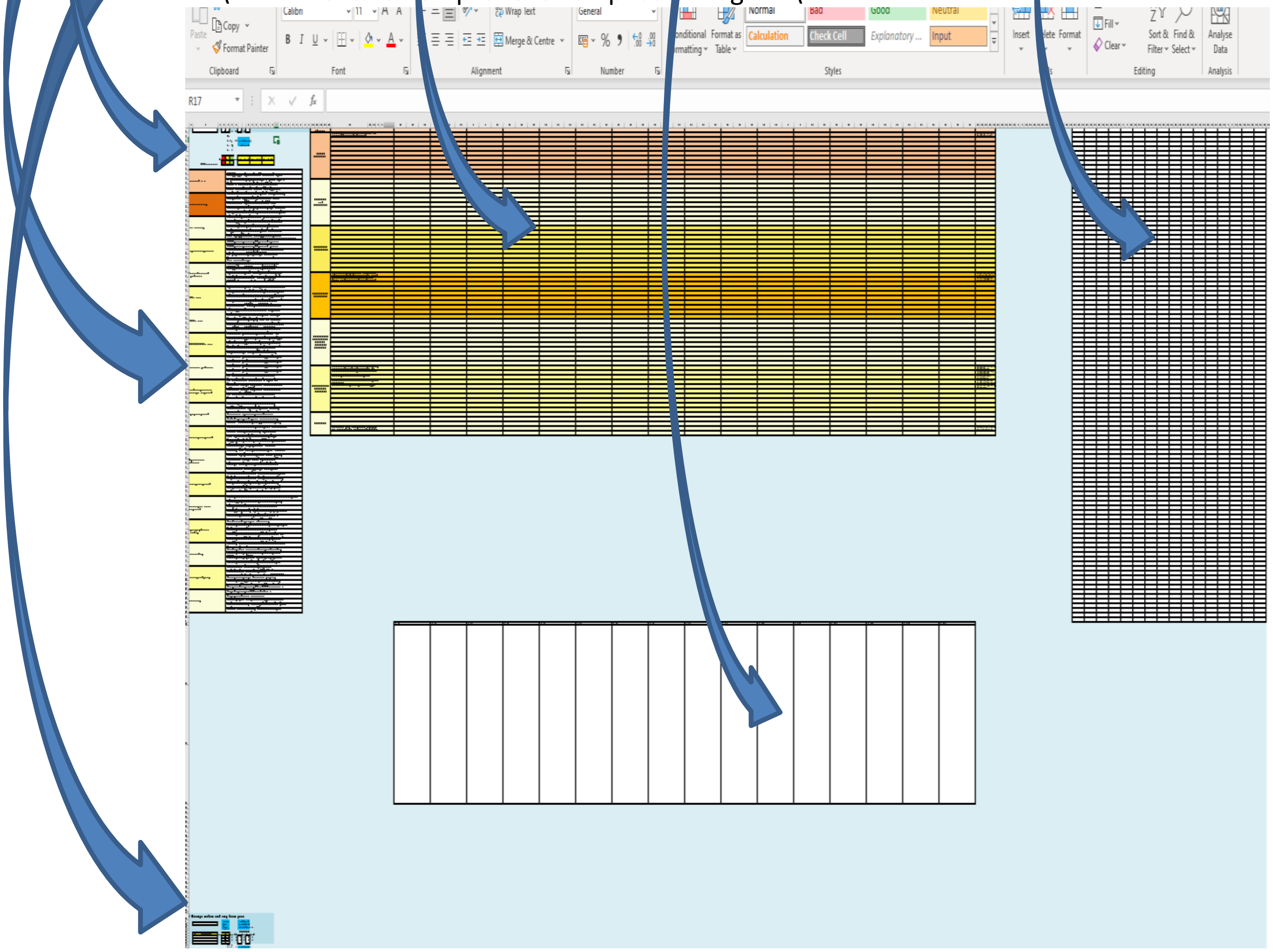

At the end of each week all notes for the 4 clinic days can be selected and copied directly to EPIC notes as a table. Clinicians may need to tidy up the appearance of

this table to separate all daily activities from one another and to remove unnecessary rows/columns.

The table should be preceded by an entry in S.O.A.P format comparable to the following:

S. <name> attended week 1 of the Queen Square ICAP (intensive aphasia treatment) at 25 Cleveland street between 9am and 4.15pm daily from Tuesday 17/3/22 to Friday 20/3/22 inclusive. They were alert and consenting to therapy at all times.

O. <Paste relevant section from recording document>

A. Mild receptive and moderate expressive aphasia

P. See in 1/52 for week 2 of the ICAP

Incident reporting

All incidents must be reported using the Datix system accessed via the trust intranet.

Clinical timetable (EXAMPLE)

**Week 1**	**Tuesday**<br>Group Room, room 2,4,12	**Wednesday**<br>Room 2, 12 and MDT (Group room also free unless booked). Room 4 pm only	**Thursday**<br>MDT room, room 2 . Room 1 pm only	**Friday**<br>Rooms 2,4 12. MDT room am. Group room pm only
9.00-10:00	INTRODUCTION GROUP<br>all therapists<br>**Group Room**	'NEWSPAPER GROUP' whole group - with …….<br>**(Group room)**	SELF-DIRECTED WORK in **MDT** ….. to supervise	ILAT group with …..<br>**(MDT)**
10:00 - 10:15	TEA BREAK	TEA BREAK	10-10:45. ILAT group with …… **(MDT)**	TEA BREAK
10:15 - 11:15	WHAT IS APHASIA and what it means	……. with Participant A		….. with participant B **(2)** **…..** with

	to you with …… **(Group Room)**. ….. and ….. admin	**(room2)** ….. with participant B and C **(MDT)** **…..**with participant D **(12)**	ADJUSTMENT GROUP (11:00-12:00) led by psychology supported by ….. **(MDT room)** ……/….. /…… admin	participant C **(12)** **…..** with participant D **(MDT)** **…..** with participant A **(4)**
11:15 - 12:15	VERB PACE with ……. **Group room**.	….. with participant A **(2)** ….. with participant B **(MDT)** **…..** with participant C **(12)** ….. with participant D **(group room if free)**		….. with participant A **(2)** ….. with participant B **(MDT)** **…..** with participant C and D **(4)**
12:15 - 13:00	LUNCH	LUNCH	LUNCH	LUNCH
13:00 - 14:00	…. with participants A and B **(group room)** **…..** with participant C **(12)** **….** with participant D **(4)**	STG GOALSETTING …… with participant A **(2)** ….. with participant B **(MDT)** …. with participant C **(12)** …. with participant D **(4)**	Travel to Queen Square – whole group activity in clinic room **(4)**	MUSEUM VISIT **(Grants Museum of Zoology)** with ……/……/…… ….. admin<br><br>Preparation and presentation of individual museum experience (group room) …… and ……
14:00 - 15:00	…… with participants A and B **(group room)** **…..** with participant C **(12)** **…..** with participant D **(4)**	….. with participant A **(2)** ….. with participant B **(MDT)** **…..** with participant C **(12)** ….. with participant D **(4)**	MEET THE DOCTORS with Neurologist and Psychologist	
15:00 - 15:15	TEA BREAK	TEA BREAK	TEA BREAK	TEA BREAK
15:15 - 16:15	…… ANNOTATION GAME **(group room)** …../……/…… admin ….. help with room and taxis at 16:10	…… DEBATING GROUP **(group room)** - ……/……/….. admin …… help with room and taxis at 16:10	MRI scans at FIL with…… supporting<br><br>….. PRONOUN GAMES - group in clinic room at Queen Square	….. 20 QUESTIONS group **(group room)** ……./……./…… admin …… help with room and taxis at 16:10
16:15	HOME	HOME	HOME	HOME

**Week 2**	**Tuesday**<br>Rooms 2, 4 and 12<br>? group room pm.<br>**…… Annual Leave**	**Wednesday**<br>Rooms 2,12 and MDT room. Room 4 pm only<br>**….. Annual leave**	**Thursday**<br>MDT room and room 2. Room 1 pm only.<br>**….. Annual leave**	**Friday**<br>Rooms 2,4 and 12. Group room pm - possibly MDT room am<br>**…. Annual leave PM.**
09:00 - 10:00	WEEKEND REVIEW + DESCRIPTION GAMES ……. in room **4**<br>……/……. admin	'NEWSPAPER GROUP' whole group - with …….<br>**(Group room)**<br>…… /….. admin	Self-directed work with ….. supervising in **MDT room.**<br>……/….. admin.<br>10:00-10:45 – ILAT group with ….. **(MDT room)**<br>…… /….. /….. admin	ILAT group in **room 4** or **MDT** with ……<br>……/…../….. admin
10:15- 10:30	TEA BREAK	TEA BREAK		TEA BREAK
10:30 - 12:15	….. with participant A **(2)** ….. with participant B **(MDT)** **…..** with participants C and D **(12)**	….. with participant A **(2)** ….. with participant B **(MDT)** **…..** with participant C and D **(12)**	BREAK<br>ADJUSTMENT GROUP 11.00-12.00 led by psychology supported by ……<br>**(MDT room)**<br>……/….. /…… admin	……. with participant A **(room2)** ….. with participants B and C **(MDT)** **…..**with participant D **(12)**
12:15 - 13:00	LUNCH	LUNCH	LUNCH	LUNCH
13.00 - 14.00	…. with participants A and B **(group room)** **…..** with participant C **(12)**<br>**….** with participant D **(4)**	….. with participants B and C **(MDT room)**<br>….. with participant A **(2)** …… with participant D **(4)**	….. dysphagia training at Queen Square<br>All therapy sessions at Cleveland Street.<br>13:00 -14:00 TRAVEL group with ….<br>……../…… admin<br>14:00-15:00 ….. with participant A **(2)** ….. with participant B **(4)**<br>….. with participants C and D **(MDT)**<br>15:15 -16:15 COUNTRY SFA Group with …… **(MDT)**	…… with participants C and D **(group room)**<br>…. with participant B **(4)** ….. with participant A **(2)**<br>….. 3month review assessment with cohort 5 participant **(12)**
14.00 - 15.00	….. with participant B **(2)** **…..** with participant C **(12)**<br>**…..** with participant D **(MDT)** **…..** with participant A **(4)**	….. with participants A and B **(MDT room)**<br>….. with participants C and D **(4)**<br>…… admin		PROCESS DESCRIPTIONS group session with …..<br>**(group room)**<br>…… /……admin.<br>….. 3month review assessment with

			……/….. admin<br>….. to help tidy room and support taxis at 16:10	cohort 5 participant **(12)**
15:00 - 15:15	TEA BREAK	TEA BREAK		TEA BREAK
15:15 - 16:15	VERB GROUP with …… **(group room)**<br>……/…… admin<br>……. to tidy room and support taxis at 16:10	TRAVEL STORIES GROUP with …… **(group room)**<br>……/…… admin.<br>……. meet with participant C and family.<br>…… to tidy room and support taxis at 16:10		TELEPHONE group with ……<br>…… to support (group room)<br>….. admin.<br>….. to tidy room and support taxis at 16:10
16:15	HOME	HOME	HOME	HOME

**Week 3**	**Tuesday**<br>Group Room, room 2,4,12	**Wednesday**<br>Room 2, 12 and MDT room. Room 4 pm only<br>**….. Annual leave**	**Thursday**<br>MDT room am (check if free pm), room 2 all day room 1 pm only<br>**….. Annual leave**	**Friday**<br>Rooms 2,4 12.<br>MDT room am. Group room pm only<br>**….. and ….. Annual leave**<br>Volunteer SLT attending
09:00 - 10:00	WEEKEND REVIEW + DESCRIPTION GAMES ……. in room **4**<br>……/……. admin	‘NEWSPAPER GROUP’ whole group - with …….<br>**(Group room)**<br>…… /….. admin	Self-directed work with ….. supervising in **MDT room**.<br>……/….. admin	ILAT group in **room 4** or **MDT** with ……<br>(supported by volunteer)<br>……/…../….. admin
10:00 - 10:15	TEA BREAK	TEA BREAK	10:00-10:45 – ILAT group with ….. **(MDT room)**<br>…… /….. /….. admin	TEA BREAK
10:15 - 11:15	….. with participant A **(12)**<br>….. with participant B **(4)**<br>….. with participant C **Group room**<br>….. with	…… with participants A and B in **MDT Room**<br>….. with participants C and D **(2)**<br>…../…… admin	ADJUSTMENT GROUP 11.00-12.00 led by psychology supported by ……	…… with participants C and D in **MDT room**<br>….. with participants A and B supported by volunteer **(4)**

	participant D **(2)**		(**MDT room)**<br>……/….. /…… admin	
11:15 - 12:15	…… with participants A and C (**12)**, ….. with participants B and D (**Group room)**<br>**……/……** admin<br>…… Next cohort BASELINE ASSESSMENT **(2)** & Psychology interview **(4)**	…… with participants C and D in **MDT room …..** with participants A and B (**2)**<br>……/……. admin		…… with participants C and A in **MDT room**<br>**…..** with participants B and D **(4)** supported by volunteer
12:15 - 13:00	LUNCH	LUNCH	LUNCH	LUNCH
13:00 - 14:00	……. Reasoning tasks in **Group Room**<br>……../…….admin<br>….. Next cohort BASELINE ASSESSMENT scoring **(2)**	FILM GROUP session with ….. in **MDT room**<br>……/…… admin	….. with participants A and B **(local café)** -<br>…. with participants C and D **(2)**<br>….. Next cohort BASELINE ASSESSMENT (**1)** & Psychology interview **(MDT)**	….. with participants A and B (**Group room)**<br>….. with participant C **(4)**<br>volunteer with participant D in **(2)**
14:00 - 15:00	TV/film clip DYNAMIC DESCRIPTION group session with …… (**Group Room)**<br>**…../…..** admin	….. with participant A (**12)** **……** with participants B and D (**MDT room)**<br>…… meeting with participant C and family (APHASIA EDUCATION) via ZOOM 14:00-15:30	Group session with …… **MDT room**<br>….. admin<br>…… Next cohort BASELINE ASSESSMENT **(1**)	RECIPE ILAT GROUP with ….. in **Group Room**<br>(volunteer supporting)<br>……/……/…… admin
15:00 - 15:15	TEA BREAK	TEA BREAK	TEA BREAK	TEA BREAK
15:15 - 16:15	TELEPHONE group session with ….. in **Group Room**<br>….. meeting with participant A and family (APHASIA EDUCATION)<br>….. to help tidy	SHOPPING GROUP session with …….<br>**MDT room**<br>……. admin<br>……. To help tidy room and support with taxis	ILAT group session with …… (**MDT room)**<br>**…….** admin<br>…… scoring baseline assessment and helping with taxis at 16:10	MUSIC group session with …… in **Group Room** with volunteer supporting<br>……. admin and helping with room/taxis at 16:10

	room and support with taxis at 16:10			
16:15	HOME	HOME	HOME	HOME

**Week 4**	**Tuesday**<br>Rooms 2, 4 and 12<br>…… **Annual leave**	**Wednesday**<br>Rooms 2,12 and Group room. Room 4 pm only<br>….. attending counselling course (all day)	**Thursday**<br>MDT room and room 2. Room 1 pm only. QS pm (MRI scans)<br>….. attending counselling course (all day)	**Friday**<br>MDT room am and group room pm - rooms 2, 4, 12.<br>….. in later at 9:30<br>Volunteer SLT attending
09:00 - 10:00	WEEKEND REVIEW + DESCRIPTION GAMES ……. in room **4**<br>……/……. admin	'NEWSPAPER GROUP' whole group - with ……. **(Group room)**<br>…… /….. admin	Self-directed work with ….. supervising in **MDT room**.<br>……/….. admin.	DEBATING GROUP with ….. **(MDT room)**<br>…… supporting volunteer re. preparation for participant D
10:00 - 10:15	TEA BREAK		10:00-10:45 – ILAT group with ….. **(MDT room)**<br>…… /….. admin	TEA BREAK
10:15 - 11:15	….. with participant A **(4)**<br>…….. with participants B and D **(2) -** telephone role-plays<br>……. with participant C **(12)**	TEA BREAK<br><br>…… with participants A and B (**group room)**<br>…… with participant C **(12)**<br>….. with participant D **(2)**	TEA BREAK (10.45)	…… with participant B **(MDT room)**<br>Volunteer with participant D **(12)**<br>…… and participant C REVIEW ASSESSMENT **(2)**<br>…… and participant A REVIEW ASSESSMENT **(4)**
11:15 - 12:15	….. with participants B and D **(2)**<br>……. with participants A and C **(12)**<br>……… admin		ADJUSTMENT GROUP 11.00-12.00 led by psychology supported by …… (**MDT room)**<br>……/….. admin	
12:15 - 13:00	LUNCH	LUNCH	LUNCH	LUNCH
13:00 -	…… with	….. with	…… with participant	….. local museum visit

14:00	participants A and C **(2)**<br>….. meeting with participant B and family 1-2.30pm (APHASIA EDUCATION) **(4)**<br>….. with participant D **(12)**	participants A and C **(2)** ….. with participants B and D in **group room**<br>…… admin	B REVIEW ASSESSMENT **(2)** at Cleveland Street (then walk with …… to QS FIL for MRI at 4pm)<br>…… with participant D REVIEW ASSESS **(1)** at Cleveland Street (then walk to QS **FIL** for MRI at 4pm)<br>….. with participants A and C in **NHNN group room** 1:30-3:00 (STORY TELLING)	**(Pollocks toy museum)** supported by volunteer ……/……admin
14:00 - 15:00	….. with participants A and C **(2)**<br>…… meeting with participant B and family 1-2.30pm (APHASIA EDUCATION) and continue with therapy 2.30-3 **(4)**, …… with participant D **(12)**	….. with participants A and C in **group room**<br>**…..** with participants B and D **(12 or 2)**<br>……. admin		…… **King and Queen pub** - ordering practice (soft drinks), discussion about museum exhibits and conversation about toys/childhood etc. (whole group)
15:00 - 15:15	TEA BREAK	TEA BREAK	TEA BREAK	TEA BREAK
15:15 - 16:15	…… ILAT group **(group room)**<br>….. (& psychology) meeting with participant D and family at 4pm on ZOOM **(12)**<br>…… helping tidy room and support with taxis at 16:15	PRESENTATIONS in **group room** with……and …..<br>Participant B 15:15 and Participant A 15:30<br>Q&A encouraged<br>…… admin and helping tidy room and support with taxis at 16:15	Participants A and C MRIs between 3.00 and 4.00 (with ……) **FIL**<br>Participants B and D MRIs between 4.00 and 5.00 (with ……) **FIL**<br>Whole group to wait at Neuroimaging centre for taxi at 5.15	Group session **(group room)** with all staff REFLECTIONS – what have we learned about each other? what's changed? what needs to change at home (new habits) and what's next?
16:15	HOME	HOME	HOME	HOME

REGULAR GROUP THERAPEUTIC ACTIVITIES – rationale and description of process

INTRODUCTION GROUP

Aim: to assess participants' level of ability providing information about themselves with and without support and to help build group rapport and familiarity. Task follows a structure (modeled by clinician) giving details of 'where I live,' 'what kind of house,' 'who I live with,' 'important people in my life,' 'pets,' 'jobs (old and new),' 'interests' 'regular activities/sports (football teams etc.)' Verbal delivery of information is encouraged supported by personal smartphone (photos/maps etc.) and gestures. Writing and drawing are encouraged as second tier methods of compensation.

TEA BREAK

Aim: to develop participants' ability to initiate requests for objects and actions (i.e. 'a cup of tea' or 'put 2 sugars in it') with attention to all necessary information using either a) a fixed phrase that becomes established beyond rehearsal and practical application or b) mixed phrases ("can I have…" "I would like…" "could you get me…") used interchangeably.

Task involves requesting objects and actions as practiced in regular constrained language games (providing an opportunity for carryover). Gesture is encouraged to assist spoken access to target verbs (i.e., put, pour stir etc.) and to give information ('a little bit' 'two' 'no milk'). Tea orders are initially prompted by direct questioning (i.e. "what would you like to drink?") and repetition of key words from the question (i.e. "like to drink…") is encouraged in participants who struggle with sentence formulation but are able to repeat accurately. The treating team aim to move away from direct requests for drinks as soon as possible and allow the situation (end of activities/familiar time) to prompt spontaneously initiated requests from one or more participants that are replicated by the remainder. Group participants recognise the challenges associated with requesting the precise set of conditions unique to their preferred hot drink and typically encourage one another – providing positive feedback appropriately.

NB. Tea preferences or common requests are often learned very quickly but the treating team do not use this learning to anticipate needs instead permitting the communication opportunity to be used to its full potential every time. This approach is employed in respect to every request opportunity across the 4 week programme (i.e., when participants arrive in the morning they may want staff to put their lunch in the fridge – staff do not anticipate this need or offer to perform the task unrequested but disregard the need until a request is initiated without any form of cue). Family members should also be discouraged from making requests on behalf of participants).

WHAT IS APHASIA – WHAT DOES IT MEAN TO ME?

Aim: Operates on the first day of ICAP to allow participants to describe their individual experiences of stroke and aphasia.

Task builds upon the biographical information provided by each individual in the first group activity of the day and allows the treating team to gauge each participants understanding of aphasia, level of awareness of their own deficits and to appreciate the impact of the condition on daily life (can also highlight barriers to progress). The group allows participants to share experiences, recognise the differences between one another and to identify common ground… as well as to draw each other's attention to services, compensatory methods and apps that they might use.

VERB PACE (Promoting Aphasic Communication Effectiveness) - gesture development

Aim: To establish the value of imitating actions not only as a useful compensatory behaviour but as a stimulus to spoken verb retrieval and to develop use of gesture assisted lexical access as a default behaviour when struggling to express a message involving an animate (i.e. non-psychological) activity.

Task uses everyday verb pictures (moderate to high frequency animate verbs). A card is issued to a single member of the group unseen by the remainder and he/she must convey 'what is happening' as completely as possible using speech primarily and gesture as the first line of compensation. Gesturing is encouraged implicitly (i.e. via expectation rather than direct cueing – clinician may look at a participant's hand) and early recognition of value to lexical retrieval is important to recruitment. Clinician's can select items for different participants based on their ability (i.e., selecting CHOPPING APPLE for somebody with pronounced word finding difficulties and selecting MEASURING FLOUR for somebody with less difficulty). The activity thrives on momentum ensuring that participants get an opportunity to communicate as many cards as possible initiating use of gesture without any form of direct or indirect cueing. Use of gesture an assistive as well as compensatory communicative method is encouraged at all times throughout the programme.

NEWSPAPER GROUP

Aim: To build independence in newspaper review – location and communication of personally interesting stories and events using keywords and any associated pictures leading to wider discussion and development of ideas.

Task initially uses multiple paper copies of daily free newspapers (such as METRO), but progresses to individually preferred news apps on smartphones OR aphasia friendly news apps (such as TALKPATHNEWS) as rapidly as possible to promote regular independent engagement with current affairs in it's most accessible and preferred format.

A clinician guides the use of 'keyword or key phrase selection and underlining/copying' strategy and rehearsal of reading aloud ahead of description to the group. Naturalistic development of ideas into broader discussion is targeted with a clinician ensuring that opportunities are not missed (seeking to elicit a conversational detour rather than suggesting it) and that all group participants are involved and have the opportunity to

contribute as much as possible. Development of peer-led turn management and inclusion/exclusion awareness is a key feature of this group.

LUNCH

Free time – participants should request their lunch from the staff refrigerator without verbal cueing or 'expectant' non-verbal cueing behaviours OR use local cafés and shops to buy their own lunch. Clinician support can be offered where problems using self service counters or managing cash are recognised - support should reduce and be removed as soon as effective strategies are in place.

ANNOTATION GAME

Aim: To encourage ideation and spoken naming within the groups' shared knowledge of a concept and its extrinsic and intrinsic components. Promotes divergent and generative thinking as well as category-based generative naming.

Task involves a clinician drawing a degraded template on a whiteboard (i.e. A very simple mammalian torso with 4 stick legs). The group should be told what the template represents (i.e. a DOG) and they should begin to name fine detail that needs to be added to the picture in order to describe the concept as fully as possible (i.e. PAWS, WHISKERS, TAIL, COLLAR etc.). Templates can be adjusted for groups with lower or higher levels of ability (including specific knowledge) – DOG/HOUSE/HUMAN BODY/CAR suit less able participants whereas INTERNAL COMBUSTION ENGINE/RADIO/COMPUTER/YACHT might suit higher level participants better. Opportunities to explore VERB description should be taken within this activity (i.e., "What does the dog do with his nose? his tail? his paws? with gesture encouraged as a means of supporting access.

DEBATING GROUP

Aim: To develop divergent thinking and reasoning skills using often controversial and emotive proposals to elicit discussion.

Task requires that the group of 4 is divided into one pairing directed to thinking of reasons FOR a given argument and another pairing directed to thinking of reasons AGAINST the same argument. Productive arguments have included 'WINNING THE LOTTERY IS EVERYONE'S DREAM,' 'OIL AND GAS SHOULD BE USED UNTIL IT RUNS OUT,' 'BRING BACK THE DEATH PENALTY,' 'FOX HUNTING IS AN IMPORTANT ENGLISH INDUSTRY,' 'FOOTBALLERS SHOULD BE PAID MORE,' 'NUCLEAR POWER IS THE ANSWER TO CLIMATE CHANGE,' 'EVERYONE SHOULD BE A VEGETARIAN.' Other arguments can be introduced – particularly if a discussion point is recognised as being close to one or more of the groups' particular interests.

Clinician guides and shapes ideas as little as possible for each pair as they ready their arguments – encourage each group to think of at least 2 reasons for their position and

write down keywords, phrases or sentences as well as they can and rehearse their statements ahead of debate. The clinician should also try to stimulate broader discussion beyond presentation of counter-reasoning.

SELF DIRECTED THERAPY

Aim: to develop participants independence accessing and progressing with appropriate level technological therapy treatments (i.e. Use of own tablet/phone encouraged with ICAP devices available for participants without own device).

Principle software packages: Cuespeak, Tactus Language, Tactus Advanced Language, Tactus Conversation bundle, Listen-In (UCL), iReadMore (UCL) Ispeakmore (UCL)

INTENSIVE LANGUAGE ACTION THERAPY (ILAT) – Constrained activities

Aim: to action-embed language use into a range of social communicative contexts using a barrier to vision as a means of constraint – placing all communicative demands on spoken language and auditory comprehension.

Task requires a sizeable table with tall obstacles placed in a cross shape to prevent participants from seeing one another's set-up, written cues or picture stimuli. Taller barriers can also limit information from facial expression and gesture, but are often challenging to construct and to keep in position. Several versions of ILAT have been employed in ICAP. The most common games have been:

a) "PREPOSITION PHRASE instruction game" – all participants have the same set of 10+ items including paired items that differ in size or colour (i.e. short pencil vs long pencil/little cup vs big cup) and take it in turns to describe a novel arrangement of items on the tabletop one item at a time. An illustrated crib sheet reinforcing the concept of locative words is provided to aid description and instructive frames such as "pick up the <> and put it <> the <>" are modelled and encouraged. The task increases participants' awareness of the need to relate an object's position to another object and there are significant demands in comprehension as well as instruction. Once the active participant has placed and described relative positioning of all items the clinician can remove the barriers and participants can compare their arrangements for accuracy.
b) "SHOPPING LIST request game" – all participants are given a shopping list for ingredients that they require to make a favourite recipe (based on personal preferences). The pictured target ingredients are issued to the other players along with a number of close foils (i.e., LEMON – lime and orange also included) and the active player must collect all the ingredients on his/her list by asking the other participants "Have you got the <>?" "Do you have the <>?" When a target is located the active participant is encouraged to request supply of the object "can you give

me the <>?" or similarly appropriate sentence frame. Participants are cued to use different sentences interchangeably across the task. The game involves significant spoken word to picture matching demands that can be lessened or increased if necessary (with fewer/more AND less distinct/more distinct foils).

c) OBJECT or ACTION picture request combined with instruction game" – participants each have an identical map (i.e., a house with defined rooms, garage, garden, road, shop, park, tree etc.) and each receptive participant is given a share of object pictures (i.e. red car, blue car etc.) and action pictures (i.e. man driving/woman driving etc.). The active participant is required to ask a selected receptive participant for a particular object or action picture (from a wanted list of 5-10 targets) and once the object is identified he/she must ask the appropriate participant to give them the picture and they must place it on there map in a location of their own choosing before describing the activity and the position as accurately as possible. Other participants are then encouraged to either draw the described event on their maps in the described location or request comparable pictures from the BANK to place on their map in much the same way. Once the active participant has requested, placed and described all items and their position on the map the clinician can remove the barriers and participants can compare their maps for accuracy.

Receptive co-players are urged to reject requests that they cannot honour (i.e. if they don't have the target item) and to ask for repetition or ask clarifying questions in case of misunderstanding. The clinician acts as a model for appropriate communication, cues repairs, and provides positive feedback. Individual therapeutic goals can be defined and difficulty levels adjusted by person-specific constraints. Verbal communication is emphasized, while gesturing is not suppressed. Therapy materials did not include any test items used in language assessments.

ADJUSTMENT GROUP (facilitated by Clinical Psychologist with SLT support)

Aim: to address what life was like before the stroke. Getting to know the 'person' before any changes. To discuss personal values; what's important and who is important currently in participants' lives. Thinking about ways of increasing the time spent with the people participants care about and doing the things that are meaningful to them (sometimes this means letting go of things or activities that are no longer possible or worthwhile). The group then concentrates on the participants' future self and what they want that to look like. Through adapting to the current self and adjusting to the changes, each participant is supported to find a renewed acceptance to what the stroke has meant and move towards not letting it define them.

Week 1 **Introduction/Who I was before my stroke**

(Before the stroke and the impact of the stroke)

- Introduction to adjustment
- Words to describe me before my stroke
- Before my stroke, I felt, my life was..
- Roles: Before my stroke, I was…
- Mindfulness exercise

Week 2/Week 3 **Who I am now/Personal values**
(What/who is important to me?)

- Social circle
- How I would describe my life right now
- Things I have learned about myself/life/loved ones
- What/who brings me joy (or I am grateful for) in my life right now
- Values & ID card

Week 4 **Living well with aphasia**

(My life in the future)

- What I think my future will be like
- What living well with aphasia means to me
- Goals in the future (value based)
- Challenges/facilitators for goals

MUSEUM VISIT
Aim: To enable enjoyment of local museums and exhibits, to develop group cohesion and to provide stimulating experiences that inspire detailed descriptions of objects or events (using advised strategies to maximize independent communication). Museum visits were typically scheduled in week 1 (to help establish key independent information gathering and reporting behaviours) and again in week 3 or 4 (to assess change in such behaviours). A set of participant self rating and clinician rating statements were answered after each visit to aid this comparison (questionnaires are available within the MUSEUM VISITS folder).
Task involved a nominated participant taking responsibility for planning and managing the excursion – deciding where to visit, how to get there and organizing the group ahead of the trip. For fully mobile cohorts the nominated participant would also bear responsibility for directing the whole group to the selected museum reading a map and issuing instructions from the back.

On arrival at the museum participants would be provided with a presentation planner and pen and be given 3 instructions

1. locate an object (or objects) that interest you
2. Take a photo of it on your phone to show it to the rest of the group
3. Try to locate important words, phrases or sentences that relate to the exhibit and copy them to the planner (in the KEYWORD section). Participants who are unable to copy or write even short keywords were permitted to take photographs of written plaques and identify/highlight keywords on their smartphones.

*Participants were encouraged to complete tasks as independently as possible but clinician support was provided where risks or additional needs were identifiable.

Upon return to the clinic participants were supported to review the information (photographic and copied) that they had gathered and combine keywords to form novel descriptions of exhibits AND/OR rehearse reading copied text as part of a presentation.

Once rehearsed, all participants take turns to present exhibits of their choosing. Presentations were often recorded for feedback and comparison purposes (week 1 and week 3/4). Any participants not consenting to video-recording (as agreed at outset of ICAP) are not filmed. All recorded footage (filmed via ipad) is uploaded to patient folders and deleted from the portable device routinely.

PRONOUN GAMES

Aim: To develop participants' ability to use personal pronouns in place of names (especially once an individual or object has been named in the first instance and is thereafter appropriately referred to via pronoun) AND to develop awareness and appropriate use of possessive pronouns.

Task involved reference to a crib sheet (gender supported personal pronouns and belongings linked to each pictured individual supported by the appropriate possessive term). Clinician models selection of a person in the room (i.e. "GARY") and then use of the correct personal pronoun to describe a characteristic (i.e. "He is tall") before describing something that belongs to him (i.e. "his hair is black"). Participants take turns describing one another before turning their attention to family members, friends and celebrities.

20 QUESTIONS

Aim: To develop logical inquiry derived from existing knowledge and awareness of high value versus low value information (salience and relevance).

Task requires pictures of many well known, varied and often contemporary celebrities of different genders, nationalities, ethnicities and backgrounds. A participant would select a pictured celebrity and keep the identity secret, fielding closed questions from

the other members of the group in turn. The objective of the game is for the unsighted participants to identify the unseen celebrity as quickly as possible. Participants that receive a “YES” response to their question can ask a further question (until they receive a “NO” response. A support tool with suggested phrases or lines of questioning can be supplied if necessary. Participants that use single words to convey questions should be supported to expand their statement to phrase of sentence level.

TV or FILM CLIP ‘DYNAMIC DESCRIPTION’

Aim: To develop participants’ abilities to perceive the core event in a short film sequence and describe the activity as fully as possible – shared focus increases awareness of predicate argument structure and thematic role assignment.

Task makes use of a screen (either ipad/laptop or wall mounted TV) with internet access. Some clips were saved within resource files but many groups accessed film clips from Youtube that reflected the preferences of the particular cohort. Familiar action films such as James Bond/Die hard etc. were especially fertile source material with distinct actions taking place in rapid succession (facilitating clinician can replay the scene and pause at very precise moments to emphasise an action or the relevance of a person or object as well as it’s thematic relationship to the verb as required). Slapstick comedy films (Laurel and Hardy, Home alone) were also well received and productive in terms of number of distinct events available for description. Sports events (i.e., highlights of recent football games) were useful for some groups – especially where individual goals related specifically to improved description of sporting events.

MEET THE DOCTORS (facilitated by Consultant Neurologist with Clinical Psychology support)

Aim: A one hour open discussion/psycho-educational session with Consultant Neurologist on a variety of topics relating to stroke (e.g. causes, prognosis and prevention) and rehabilitation (e.g. recovery trajectories, plasticity)

WEEKEND REVIEW

Aim: For all participants to offer as much detail about their weekend activities as possible without direct questioning – use of smartphone cameras and supportive materials is encouraged

RECIPE GROUP

Aim: To use a staged approach to thinking about a familiar recipe to aid step by step ideation and verbal description to the other members of the group.

Task is often employed as either 2 compatible pairs working on the same recipe OR 2 compatible pairs working on different recipes. Occasionally all participants were sufficiently able to work alone preparing a description of their own recipe and preparation process.

Step 1. Participants to think about the ingredients required for their recipe – these should be written down in a group for later reference (paired participants can help one another with this task but it is advised that the responsibility is shared – not one participant doing all the writing).

Step 2. Participants to think about the equipment needed for their recipe – again, these should be written down in a group for later reference.

Step 3. Think about process – try to describe each step of preparation using the specific verb relative to SUBJECT and OBJECT (i.e. "Use a colander to STRAIN the peas")… gesture is encouraged as a means of supporting lexical access.

Step 4. Once process has been established and rehearsed the individual or pair can describe their agreed step-by-step instructions to the rest of the group (sharing the responsibility as much as possible).

TRAVEL STORIES GROUP

Aim: To elicit high quality and stimulating narratives about positive and negative travel experiences using discourse strategies learned in individual therapy

Task requires a map of the world and modeled examples of positive and negative experiences. Participants can use the map to set context and can use other compensatory methods to elaborate. Other participants should be encouraged to ask questions and to contribute to broader discussion generated by unusual or inspiring stories.

PROCESS DESCRIPTIONS GROUP

Aim**:** To develop ideation skills in the conceptualisation and description of familiar multi-step tasks (i.e. changing a tyre on a car/making a cup of tea/travelling through an airport).

Task is often completed collaboratively with all group participants contributing to the same description. The activity can often benefit from key written words arranged in order of process (as they are identified) – can reinforce ideation of the next stage.

MUSIC GROUP

Aim: To share enthusiasm for and knowledge of artists and/or musical works via supported independent research, preparation and rehearsal of key words, phrases or sentence/paragraph level description.

Task results from individually supported preparation (in single or paired sessions), using

personal smartphones or clinic computers to research favourite songs or artists ahead of a short presentation culminating in the selected piece of music being played to the group. Other participants can then comment on the song describing their personal sentiments, how it made them feel and any other information (written adjectives can be provided for selection - from a pool on the tabletop).

PRESENTATIONS

Aim: To develop participants ability to tell personal narratives and share their identities through personal stories and interests.

Task required keyworker supported development of a powerpoint or handwritten presentation with as much independent contribution as possible (i.e. collecting photographs and information from home and the internet); developing a structure, making decisions on detail (what to add in/what to omit) and to rehearse ahead of formal presentation to the group.

APHASIA EDUCATION – optimisation of systemic communication

Aim: For people with aphasia to better understand their condition, what helps to promote improvement/what hinders further recovery and to communicate this learning to selected people in their system (family, friends, colleagues etc.)

Task involves keyworker led tailoring of a ‘My aphasia and how to support me’ powerpoint presentation (see appendix D - page 59). The participant makes decisions on content (based upon their experience and supported understanding of their deficits and what helps). The template presentation includes content that may not apply to particular individuals and this should be deleted as appropriate. Similarly, advice regarding ‘what helps’ and ‘what doesn’t help’ requires individual selection.

Once a participant’s presentation is complete the person with aphasia can liaise with people they want to attend (either an in-person education session OR a remote Zoom session – with support from keyworker as required), to schedule a date and time for the education session. The presentation should be jointly facilitated by the keyworker and the participant – the person with aphasia should lead as many aspects of the session as possible and the clinician should aim to ‘fill-in’ ‘add rationale’ rather than dominate the session. Participants with more severe aphasic profiles will of course require more substantial support, but the presentation should always maintain the character of participant led or shared delivery.

Following education sessions an individual’s presentation slides may be shared with the participant to distribute as they see fit. Wherever this may not be possible keyworkers can share the slides directly with carers or nominated family members for broader dissemination.

CARERS' CAFÉ – Psychology led forum for nominated carers of people with aphasia (supported by psychology assistant or research assistant)

Aim: To support the carers of people with aphasia, in order to adjust and adapt to the changes they're experiencing because of the stroke. The group follows the principles of acceptance and commitment therapy. Themes include letting go of the struggle, increasing mental flexibility, unhooking from the pain and loss, and acknowledging the strain that sometimes accompanies this role.

DURING ICAP Face to face for 1 hour weekly (4 sessions)
Aim: To provide holistic interventions informed by initial systemic assessment with carer(s)

FOLLOWING ICAP Remote Carers' café for ongoing adjustment for 1 hour weekly (4-6 sessions)
Aim: Establishing a legacy of peer-support

Intervention:

- Record pre/post outcome measures (e.g. care-giver burden, mood, self-efficacy)
- Set individual goals for carers (GAS - relationship goals are key)
- Therapeutic Intervention
- Psycho-education (about stroke and aphasia)
- Acceptance and Commitment therapy/group psychotherapy to re-establish carers' identity in their new role
- Encouragement of peer-support (Klonoffand Koberstein, 2010)
- Encouragement of continued support during adjustment period

<u>REGULAR INDIVIDUAL/PAIRED THERAPUTIC ACTIVITIES</u>

**Cognition (attention and memory)**	**CLQT Cognitive Linguistic Quick Test assessment (symbol cancellation):**
	**Shopping game - generation of items and recall of multiple items in series:**
	**Simple OR challenging alphabet games (simple - naming items A-Z within a category using graded support) OR challenging - naming items A-Z switching between 2 categories):**
	**Auditory memory tasks (either non-verbal recall OR recall via repetition) single syllable words/multisyllabic words/digits or short sentences in series:**
	**Mathematics – lower-level arithmetic and matching spoken numbers to digits (extending to moderate/higher level tasks if**

	indicated):
**Cognition (executive functioning)**	Self-directed work on non-verbal problem solving and strategy formulation skills - mazes/colour trails/block design/dominoes/connect 4/draughts/chess etc:
	Divergent naming - generating distinct items within a given category (i.e., RED ="post-box" and "ladybird"):
	Guess who?' with varied celebrity faces (small set to large set) - focus on strategy & question formulation for rapid identification:
	Reasoning and debating skills - generating arguments for and against a motion with graded support (controversial topics/who should survive?/balanced choices i.e., country vs city living?):
	Clue generation task (revealing an unseen object or picture in 3 stages (hard clue/medium clue/giveaway):
**Conceptual semantic integrity**	Odd one out (picture) identification at simple level (4 choices - superordinate level of distinction) extending to moderate (5 choices at coordinate level) and advanced level (6 choices at close coordinate or subordinate level of distinction):
	Matching picture pairs by close association (i.e., HAMMER - NAIL) - small set of largely distinct pairs extending to larger set (i.e., 30x pairs) including foils:
	Picture sorting by category - access to conceptual attributes and intrinsic properties via decision making 'does the given picture fit the category?' EASIER activities focused on explicit characteristics (i.e., BIG or SMALL?) extending to HARDER activities focused on implicit characteristics (i.e., SWEET vs SALTY?):
	Supported exploration of conceptual semantic features (i.e., via drawing - adding detail to a template… extending to annotation):
	World map supported activity - matching pictured or spoken ANIMALS/FOOD/LANDMARKS/CLOTHES/CELEBRITIES/EVENTS to associated countries:
**Event perception /message conceptualisation**	EASY picture sequencing (3-4 pictures with explicit cues):
	HARD picture sequencing (6-8 pictures with implicit cues):

	Action identification tasks ranging from overt ANIMATE actions (i.e., kicking) to covert PSYCHOLOGICAL actions: (i.e., remembering):
	Complex picture description - grading item relevance & identification of movement, intention or potential with focus on access to animate and psychological verbs to describe inter-relation/interaction:
	Treatment of underlying forms:
Lexical semantics (spoken and written word comprehension)	Matching spoken or written word to a given category (i.e., TOOLS), attribute (i.e., HEAVY), or property (CONDUCTIVE) - exercises range from EASY to HARD level:
	Sorting spoken or written words by category (difficulty level ranging from easy superordinate choices to challenging close coordinate or subordinate choices):
	Spoken word to picture matching - difficulty level ranging from easy (i.e., 2 choices at superordinate level of distinction) to hard (i.e. 12+ choices at close coordinate or subordinate level):
	Written (or spoken to written) synonym matching ranging from EASY (high frequency + high imageability) to HARD (low frequency + low imageability) level - graded (choice of 2/3/4/5):
	Written word to CATEGORY association decision – circling associates and crossing out non-associates (mix of close, moderate and distantly associated words all word classes, levels of frequency and abstraction + non-associated foils:
VERB semantics	Spoken word to picture matching - VERB PICTURES - difficulty level ranging from easy (i.e., 2 choices at high level of distinction i.e., EATING vs KICKING) to hard (multiple choices at low level of distinction (i.e., HOPPING vs JUMPING vs SKIPPING):
	Verb generation based on noun exploration (i.e., using a real object to explore different means of manipulation and application) - associated actions (i.e., KNIFE - "hold, cut, slice, chop, spread, wash, dry, put away, point etc.):
	Use of real objects (AGENT and PATIENT) to support access to gesture (i.e., replication of action beyond real object use and rehearsal):
	Verb specificity task - selecting 'best fit' written VERB to describe action of a given AGENT on a PATIENT (tasks range from EASY 2-3 choices to HARD 4-10 choices:

	Written VERB synonym matching at either 2 or 3 choice level (reducing level of distinction):
ADJECTIVE semantics	Matching spoken or written adjectives to a stimulus picture or word:
	COLOURS - spoken or written word to picture matching (5 distinct choices to 15+ less distinct choices):
	Generating clues (i.e., sizing, shaping, movement imitation, noise imitation, word associations, relevant colours etc.) and combing in support of stimulus picture communication:
	Grading value of descriptive words to identification (via PACE activity… HARD clue, MODERATE clue, GIVEAWAY clue - executive demands):
	Describing character (use of adjectives to define historical or fictional personalities) - selecting appropriate adjectives from multiple options AND/OR generating appropriate descriptors (i.e., FAGIN - "miserly"):
PRONOUN and PREPOSITION semantics	Pronoun comprehension or production task - 20 mixed male/female pictures to support correspondence and accurate production (self-monitoring and repair ability):
	Pronoun task - using personal pronouns with pictorial support to describe pictured male/female characteristics and features:
	Pronoun task - using personal and possessive pronouns to describe other group participants attire, physical features and characteristics:
	Rehearsal of locative concepts aided by crib sheet (conceptual reference) - use of 2 distinct high frequency objects (i.e., book + pen) to comprehend and describe positional relativity:
	Preposition barrier game - issuing instructions and following complex locative instructions in paired or group object placement/orientation task (i.e., "put the … under the …"):
Spoken sentence level comprehension	2 Keyword comprehension task - identifying pictured target from reversible options - spoken active sentence stimulus:
	2 Keyword comprehension task - identifying pictured target from reversible options - spoken passive sentence stimulus:
	3 Keyword comprehension task - identifying pictured target from reversible options - spoken active sentence stimulus:
	3 Keyword comprehension task - identifying pictured target from reversible options - spoken passive sentence stimulus:
	Use of strategy to improve comprehension of embedded and post-modified clause:

**Auditory perception and word recognition (lexical access) + phonological output/motor speech**	**Assessment of auditory discrimination - minimal pair same/ different decision? (shielding articulation):**
	**Assessment for word deafness - real word versus non word decision (words from all classes with varied legal and non-legal morphology):**
	**Assessment for word deafness - real word versus non word decision (words from all classes without non-legal morphology):**
	**Written word rhyme judgement (paired 'same/different'? Or odd one out) at less distinct/HARD level:**
	**Written word rhyme judgement (paired 'same/different' decision or odd one out) at distinct EASY level:**
**Single word naming (expressive speech)**	**Phonological feature analysis (picture decision: long word/short word identification, syllable decision first/last sound to picture matching:**
	**Confrontational naming via errorless phonemic cueing approach (nouns/verbs - primed or un-primed):**
	**Association naming - identification of strategy to assist verbal fluency:**
	**Word finding strategy work - e.g., circumlocution, self-cueing:**
	**Total communication strategy training - gestures, drawing, writing etc:**
**Sentence level production (expressive speech)**	**VNEST (Verb Network Strengthening Treatment) - subject - verb - agent:**
	**Freeze frame description - use of TV clips / film clips to focus on event perception 'what is happening now?':**
	**TV/Film clip aided description - focus on 'what is going to happen?' and 'what happened?' - development of awareness of tense:**
	**Mapping therapy (reordering sentences - switching object and subject to develop grammatical flexibility):**
	**PACE activities (promoting aphasic communication effectiveness) - describing unseen pictures ranging from simple to complex event (level of detail):**
**Self monitoring/repair skills - perseveration reduction**	**Development of meta-cognition (recording communication performance and replaying - client self-rating versus clinician rating):**
	**Picture naming or picture description as a means of improving self-monitoring of output accuracy (no. of errors recognised: no. of errors repaired):**

	Perseveration management - naming of unrelated, phonologically distinct pictures in randomised order with focus on recognising perseverative utterance + suppression (use of a strategy to target lexical form of stimulus):
	Practising self-monitoring and repair skills in discourse/real-world communication:
	Education of family/carer re. supporting self-monitoring and perseveration reduction skills:
**Discourse level performance (expressive speech)**	Procedural narratives – issuing instructions (i.e., how to make a cake, change a tyre, build a house, travel to the moon?) via identification of key vocabulary, key actions and essential steps (encouraged use of determiners and conjunctions in description):
	WH questions (beyond reinforcement of question to concept correspondence and using support tool) - asking and responding to biographical questions/discussion of historical events within a paired or group activity:
	STORY TELLING – using NARNIA and mind-mapping approaches to organise information when recounting events (BEGINNING setting context people/place/time WHAT WAS HAPPENING & WHAT HAPPENED NEXT):
	Describing events from sports events (football matches), well known films or books (scene by scene) – organising information in series:
	Delivering a prepared/rehearsed presentation to the group about interests, past job, travel etc.+ fielding and answering questions:
**Functional / participation level performance (expressive speech)**	Ordering drinks/food in a café or restaurant beyond identification of strategy and rehearsal:
	Telephone role playing BASIC (requesting services using preparation and rehearsal of greeting, key questions, and anticipated information including name/address/phone number OR ADVANCED (paying for goods, reading credit card details aloud reliably, complaining about poor service etc. beyond rehearsal):
	Asking for assistance (i.e., where are the…?) or directions (i.e. the way to…?) within community based tasks:
	Directing group (reading map and adhering to pre-planned route) using spoken instructions from the rear during community-based activities:

	Recounting details of an experience (i.e., British Museum visit) - participants encouraged to record key words/key information about points of interest (written words/sentences, photographs, rehearsal of key vocabulary) and rehearse communication of the information to the group:
Single word/ sentence / paragraph / text level reading	Letter by letter reading using cross modality cueing:
	Reading aloud using ORLA (Oral Reading for Language in Aphasia) approach:
	Reading 2 letter function word lists accurately 'against the clock' - how many correct in a minute OR whole list read accurately in 'how long?' (seconds):
	Reading 3 letter function words 'against the clock' - how many correct in a minute OR whole list read accurately in 'how long?' (seconds):
	Reading verbs with differently inflected endings 'against the clock' - how many correct in a minute OR whole list read accurately in 'how long?' (seconds):
Functional reading	Reading menus/takeaway leaflets or TV schedules accurately (showing understanding):
	Reading texts/short emails accurately (showing understanding):
	Matching newspaper headlines to pictures/captions/text at EASY (overt cues) to HARD (implicit information) level:
	Reading Newspaper stories at multiple short paragraph level employing strategy of circling keywords, underlining difficult to access words and identifying verbs to assist comprehension of meaning. Verbal summary issued beyond preparation:
	Reading aloud (either reading to children, reading short articles of interest or reading poems/jokes etc.):
Single word /sentence level writing (and spelling)	Written letter fill - salient omitted letter in short high frequency words with or without options (i.e., Target CAT - _ A T with letter options P or C):
	Written letter fill - less salient omitted letters in longer lower frequency words with or without options (i.e. Target KANGAROO - K A _ G A _ O O with letter options S, N, T and R):
	Identifying correct spelling (from x3 close foils) - complex spellings:
	Metacognitive approach - writing discourse after analysing example texts and identifying "beginning, middle and end" -

	**e.g., as in narrative therapy approach:**
	**Written picture description using SV and SVO sentence frames:**
**Functional writing**	**Writing a short greeting in a birthday card (with name):**
	**Writing a postcard activity (from imagined exotic locations):**
	**Developing text/email writing ability - extending production and working on structure - compensatory methods (speech to text? Picture texting? Use of emojis?):**
	**Presentation development - independent use of computer to produce a Powerpoint or word document to support a presentation related to a chosen theme:**
	**Curriculum Vitae (CV) development and or covering letter writing using computer (word etc.):**

REFERENCES AND OTHER INTERVENTIONS:

- Semantic feature analysis (Boyle et al American Journal of Speech-Language Pathology. 1995;4:94, Efstratiadou et al. Journal of Speech, Language, and Hearing Research. 2018;61:1).
- Semantic links (Bigland et al. Semantic Links. 1992)
- Speech sounds on cue (with functional whole words as the target) (Bishop. Speech Sounds on Cue. 2011)
- Semantic approaches: semantic feature analysis with semantic distinctions (Boyle 1995 op. cit., Efstratiadou 2018 op. cit.) semantic-phonological word maps (incorporating semantic feature analysis and phonological component analysis based on the individual loci of breakdown)
- Intensive language-action therapy (ILAT) (See – ILAT: The methods Stephanie Difrancesco, Friedemann Pulvermüller & Bettina Mohr)
- Phonological and semantic cueing hierarchies (Linebaugh et al. Aphasiology. 2005;19(1):77)
- Barrier games using functional words e.g. Promoting Aphasic's Communicative Effectiveness (PACE) (Davis, Aphasiology. 2005;19(1):21, Edelman. Promoting Aphasics Communicative Effectiveness 1987)
- Compensatory strategies such as total communication, drawing (Byng et al. International Journal of Language and Communication

Disorders. 1999;34:265, Sacchett, et al. in The Aphasia Therapy File Volume 2 2007), and gestural facilitation (Raymer et al. Neuropsychological Rehabilitation. 2011;22:235)

- Letter by letter reading using cross modality cueing (e.g. saying the letter sound, letter name and copying) (Nitzberg Lott et al. Aphasiology. 1994;8(2):181)
- “Sounding out” techniques such as re-learning lettersound correspondence (GPCs)(Partz. Cognitive Neuropsychology. 1986;3(2):149)
- Whole word recognition tasks e.g. single written word to picture matching, or recognition and reading of irregular words (Coltheart in Seron et al. Cognitive Approaches to Neuropsychological Rehabilitation 1989)
- Homophone judgment / identification (Scott et al. Aphasiology. 1989;3(3):301)
- Phoneme-Grapheme Re-Training (Hillis Trupe in Brookshire. Clinical Aphasiology. 1986, Kiran. Aphasiology. 2005;19(1):53)
- Mapping Sounds to letters using a key word (Beeson et al. Aphasiology. 2000;14:551)
- Written Naming Tasks (Hillis et al. Brain and Language 1991;40(1):106)
- Anagram and Copy Treatment (ACT) (Beeson et al. Aphasiology. 2002;16:473)
- Copy and Recall Treatment (CART) (Beeson et al. Journal of Speech, Language, and Hearing Research. 2003;46(5):1038)
- Self-dictation strategy (Pound. Aphasiology. 1996;10(3):283)
- Repetitive reading aloud at sentence level to improve familiarity and comprehension such as Multiple Oral Reading (MOR) (Kim. Contemporary Issues in Communication Science and Disorders. 2010;37, Tuomainen et al. Aphasiology. 1991;5:401) and Oral Reading for Language in Aphasia (ORLA) (Cherney et al. Rehabilitation Literature. 1986;47:112)
- Strategy based treatments such as using a place keeper, focused attention, reading and re-reading. (Cocks et al. Aphasiology. 2013;27:509, Gold et al. Reading Psychology. 1984;5:65, Lynch et al. Asia Pacific Journal of Speech, Language and Hearing. 2009;12(3):221, Webster et al. Aphasiology. 2013;27(11):1362)
- Reading & Summarizing tasks such as Attentive reading and

constrained summarization (ARCS) (Rogalski et al. Aphasiology. 2008;22:763)

- VneSt (Edmonds op. cit. 2011)
- Reading for inference (Baretta et al. Psychology & Neuroscience. 2009;2:137)
- Supported reading comprehension - incorporating aphasia-friendly text supports such as pictures or underlining key words (Dietz et al. Aphasiology. 2009;23:1053, Knollman-Porter et al. Topics in Stroke Rehabilitation. 2016;23(4), Rose et al. Aphasiology. 2003;17(10),:947)
- Compensatory strategies such as using text to speech apps (Harvey et al. Communication Disorders Quarterly. 2013;35)
- Written picture description using sentence verb object structure (Salis et al. APHASIOLOGY. 2010;24(9):1051)
- Compensatory strategies such voice recognition or “speech to text” (Bruce et al. International journal of language & communication disorders. 2003;38:13, Estes et al. Aphasiology. 2011;25(3):366, Thiel et al. Aphasiology. 2015;29(4):423)
- Answering wh-questions using a modified treatment of underlying forms approach (thompson op. Cit. 1993)
- Metacognitive approach – writing discourse after analysing example texts and identifying “beginning, middle, end” – such as the narrative therapy approach described in (Whitworth op. Cit. 2015)
- Mapping therapy approaches targeting comprehension (Schwartz et al. Aphasiology. 1994;8(1):19)
- Verb Network Strengthening Treatment (VNeST) (Edmonds et al. American Journal of Speech-Language Pathology. 2011;20:131)
- Response elaboration training (RET) (Wambaugh et al. American Journal of Speech-Language Pathology. 2013;22(2):S409)
- Verb Network Strengthening Treatment (VNeST) (Edmonds op. cit. 2011)
- Script training (Goldberg et al. American Journal of Speech-Language Pathology. 2012;21:222)
- Mapping therapy (production) (Rochon et al. Neuropsychological Rehabilitation. 2005;15(1):1, Schwartz op.cit. 1994)
- Treatment of Underlying Forms (Thompson et al. Aphasiology. 1993;7(1):111)

- Structured mind mapping approaches for expository narratives highlighting "beginning, middle and end" such as that described by (Whitworth et al. Aphasiology. 2015;29(11):1345)
- Modified response elaboration treatment for personal recounts (Wambaugh op. cit. 2013)
- Total communication (Lawson op. cit. 1999)
- Conversation partner training with friends, carers and or family involves using video for PWA and their family/friends to identify the most effective strategies to support communication (Simmons-Mackie et al. Arch Phys Med Rehabil. 2016;97(12):2202)
- Tree of Life narrative therapy framework (Ncube. International Journal of Narrative Therapy & Community Work. 2006;(1):3) used to support individuals to:
- Psychotherapeutic support group focusing on adaptation to major life changes, self-management and emotional wellbeing (Off et al. Topics in Language Disorders. 2019;39(1):5)

GOAL SETTING, REVIEW AND SCORING (Goal Attainment Scaling GAS)

Short-term goals are set on day 2 of the ICAP and uploaded to the relevant area of the participant's recording document (see page 18) soon after with all weightings included. The spreadsheet has the GAS formula preloaded so that baseline score, final score and change score will auto-generate as BASELINE and ACHIEVED scores are amended. Clinicians must ensure that the ACHIEVED score replicates the BASELINE score initially – until such point as there is progress or at the point of final review (this enables the formula to work without problem).

1. Identify the goals Meet with the participant - seek to identify the main problem areas and establish an agreed set of priority goal areas for achievement by the end of the programme. A goal setting support tool (see appendix C – page 58) may help with this process. Set goals should follow the SMART principle – that is, they should be Specific, Measurable, Attainable, Realistic and Timely.

2. Weight the goals Assign a weight to each goal using the table below. Weight = importance x difficulty. Importance and difficulty may each be rated on a 4 point scale:

0 = not at all (important) 0 = not at all (difficult)

1 = a little (important) 1 = a little (difficult)

2 = moderately (important) 2 = moderately (difficult)

3 = very (important) 3 = very (difficult)

*Goals rated as “not at all” important will not be selected.

3. Define expected outcome The “expected outcome” is the most probable result if the patient receives the expected treatment. Define also the levels for “somewhat less” and “much less” “somewhat more” and “much more” These are defined by the team and should be as objective and observable as possible. This process also provides an opportunity to negotiate with the participant if they have unrealistic expectations.

Goal Attainment Scaling:

**GOALS set (date):**	
**GOALS final review (date):**	

0 = not at all
1 = fairly
2 = very
3 = extremely

much better = 2
somewhat better - 1
expected outcome = 0
somewhat worse = -1
much worse = -2

**Patient stated goals:**	**Agreed SMART goal description:**	Importanc	Difficulty	Weight	WSq	Baseline	W x base	Achieved	W x Ach
		1	3	3	9	-1	-3	0	0
		1	3	3	9	-2	-6	0	0
		2	3	6	36	-2	-12	0	0
			SumW	12	144		-21		0
			Sum (Wsq)		54				
			Factor	81					
			Sqrtfactor	9.0					

SCORES

	Baseline	Achieved	Change
GAS calculation	26.7	50.0	23.3

Achieved post ICAP	Achieved at 3 months	Achieved at 12 months

Medium term goals

These goals are identified on day 15 or 16 of the ICAP (at the point of short term goal and assessment review). Most goals will either relate to short-term objectives and seek to target generalisation of new skills to participation and activity, but some will seek to establish new behaviours that represent a significant change in behaviour. Each participant should set at least 1 medium term goal with 2-3 encouraged. Patient stated goals and the keyworkers SMART interpretation should be uploaded to the relevant formula in the participant’s recording document (see page 19) and weighting scores should be added as per the instructions for short-term goals. Medium term goals are reviewed at the 12 week post-ICAP appointment and scores are amended according to satisfaction of the criteria stipulated within the SMART descriptor. Achieved score and change score for medium term goals should be uploaded to the outcomes spreadsheet along with the SMART medium term goal descriptions and confirmation of status (i.e., NOT ACHIEVED, PARTIALLY ACHIEVED, ACHIEVED).

Long term & Economic goals

Long term & Economic goals should be identified and agreed at the end of the ICAP but can be created or altered at the 12 week review assessment if necessary. Participants should be supported to identify objectives that represent a highly desirable change in the manner in which they live (i.e. increased independent activity, increased socialisation or participation) and should where possible target an outcome with tangible economic value. This can be either paid or unpaid (voluntary) occupation on the part of the ICAP participant or a carer previously unable to work due to the demands of their care role. Economic value can also be argued as a by product of increased community engagement and use of services.

Long-term goals and economic goals were reviewed at the 12 week post assessment in ICAP year 2 but were not scored using GAS. A record of each goals wording and status was uploaded to the outcomes recording document.

DISCHARGE REPORTING
Template located at: M:\ICAP Intensive Comprehensive Aphasia Program\ICAP YEAR 2\Patient info and documentation YEAR 2\ICAP 2022 Patient folders\MASTER template new patient folder

To be completed within 2 weeks of a participants' completion of the ICAP and to include:

- a summary of the programme
- a brief overview of interventions
- a summary of goals (short term goals and outcomes + agreed medium term/long term goals for attainment)
- a summary of review assessment findings
- recommendations and advice for the person with aphasia and their family

The discharge report is written for all audiences that are likely to gain from comprehensive information (i.e. the person with aphasia / their family / their community or private SLT / other health professionals including GP) – it should provide a valuable level of detail accordingly but key words can be emboldened to support the primary recipient's ability to access the information.

For example discharge report - please see Appendix A.

APPENDICES:

A. Discharge report

Intensive Comprehensive Aphasia Programme @ Queen Square

A.

Private & Confidential

Date:

Dear

**Patient Demographics**	**GP details**
**Name:**<br>**D.O.B:**<br>**Gender:**<br>**MRN number:**<br>**NHS number:**<br>**Address:**<br><br><br>**Telephone number:**	**GP practice identifier:**<br><br>**GP address:**

Thank for attending the Queen Square Intensive Comprehensive Aphasia Programme between the dates of ……and ……(inclusive).

The 4 weeks included the following:
Group work – improving conversations, telephone role plays and community-based activities

Individual speech and language therapy sessions

Paired speech and language therapy sessions

Education session with Speech and Language Therapist ………

Stroke information session with Consultant Neurologist ………

Adjustment group with Clinical Psychologist ……..

The adjustment group addressed what life was like **before** the stroke. This was a way of getting to know the '**you'** before any changes.

We then went on to discuss your **values**; what's important to you and who is important to you, currently in your lives. We thought about ways of **increasing the time you spend with the people you care about** and **doing the things that are meaningful to you.** Sometimes this means letting go of things or activities that are no longer possible or worthwhile.

We then concentrated on your future self and what you want that to look like. Through **adapting** to your current self and **adjusting to the changes**, we can find a **renewed acceptance** to what the stroke has meant for you, and hopefully work towards not letting it define you.

- Carer's café - support for carers with Clinical Psychologist ……….

Your partner attended the Carer's café. This group was to support the **carers of people with aphasia**, in order to adjust and adapt to the changes they're experiencing because of the stroke. This group follows the principles of **acceptance** and **commitment therapy**. Themes include letting go of the struggle, increasing mental flexibility, unhooking from the pain and loss, and acknowledging the strain that sometimes accompanies this role.

Your goals:

Goal (examples)	Time frame	Achieved or ongoing
"Speaking better"<br>To spontaneously tell a story with at least one sentence (SV or SVO) - no cueing - on at least one occasion by end of ICAP	4 weeks	Achieved<br><br>You spontaneously produced "I used to play piano" and "I bought a…<table – from furniture shop>"
"Read better"<br>To be able to match 10x 4-6 word headlines to the correct corresponding picture (choice of 10) with at least 70% accuracy by end of ICAP	4 weeks	Partially achieved<br><br>Task relied upon understanding at least 2 keywords from each headline (i.e. picture options shared items in different contexts) – you progressed from matching 1/10 at the beginning of the programme to matching 5/10 with confidence.
"To write better" (using ipad/computer)<br><br>To select and copy words, phrases and questions from a selection of scripted options into an email to my dad and send independently by end of ICAP	4 weeks	Partially achieved<br><br>You were able to independently type a well-structured message (via copying) on her ipad but needed assistance to add in the contact email address and

		send.
To regularly write emails and texts to family/friends via copying script method and to send independently	3 months	Ongoing
To access and use the camera function of ipad reliably and to regularly (more than 3 times per week) forward everyday pictures to contacts (independently) as a means of starting conversation.	3 months	Ongoing
To access a local shop with minimal to no support (i.e. via a powered chair – self driven) and ask for something from a shelf (beyond rehearsal), pay for it independently with cash, check the change and take home successfully	1 year	Ongoing

Summary:
You were very motivated during the programme and made the following improvements:

Goal achievement – you made good progress on your goals:

- You are **getting better** at combining the PERSON and the ACTION when **describing events** – you benefit from **using gesture** to help retrieve the action word and you are doing this spontaneously most of the time.

- You have a **more accurate understanding of the meaning of words** (both spoken and written) – you can recognise associations and access intrinsic information more reliably which helps you problem solve communication of your message better and has improved the quality of your gesture and drawing. Understanding of spoken language remains better than for written language but you are improving – you have not yet managed to achieve your goal of matching 7/10 2 keyword headlines to the correct pictures.

- You are **able to copy words, phrases** and short sentences accurately (including typing them out on keyboard). You have shown ability to compose messages from optional phrases and sentences and you have the potential to send these messages to your contacts independently. You have also developed the ability to **use the camera** on your **ipad** and to **take photographs** – it is hoped that you can begin to forward these pictures as a means of communication.

Assessment gains (reassessment using the Comprehensive Aphasia Test)

Semantic memory – your score increased from 8/10 to 10/10
Initially your ability to perceive associations between objects, people, places, actions and descriptive concepts was unreliable – particularly for weaker associations.

You are now able to **access more information about the things you are thinking about** (including information that is implicit - not so visible or recognisable). More work on this area and improved access to implicit information will further benefit your language functioning and your ability to problem solve communicating your message by other means (including drawing).

Gesture – your score increased from 1/10 to 12/12

Your ability to generate **gestures** that accurately resemble the target action has **improved greatly** due to better access to the semantic information and 'idea' of object use. You are now using gesture spontaneously to support your message and you can sometimes retrieve spoken word for actions that you imitate very accurately.

Understanding spoken language – your **understanding of spoken words improved** from 17/30 to 24/30 and your understanding of spoken sentences improved from 15/32 to 20/32.

Understanding written language **–** your **understanding of written words** improved from 13/30 to 16/30 and your understanding of written sentences increased from a score of 4/32 to a score of 13/32. Your mistakes at single word level were all related to close semantic alternatives (i.e. selecting pictured TULIP for written stimulus "ROSE").

Spoken language – Your score for **naming objects improved** from 0/48 to 2/48.
You named the practice item "car" and the first test picture "knife" but then struggled to get the words for the next 8 items. This resulted in discontinuation of the subtest (from item 9 of 24).
Your **ability to name actions** (verbs) **increased** from 0/5 to 3/5. You were seen to use gesture (imitation of actions) spontaneously for all 5 verbs.

Your **ability to repeat** spoken words **improved** from a score of 14/30 to 26/30. You benefit from being able to see the speaker's mouth in repetition – this helps you to correctly replicate the mouth shapes and movement patterns involved.

Your ability to read aloud improved marginally from a score of 0/48 to a score of 2/48

Written production – your ability to copy letters accurately improved from a score of 13/27 to 27/27. All scores related to written naming improved marginally

Communication confidence – your **self-rating for communication confidence** (using the CCRSA) **increased** from 0/100 before the programme to a score of 55/100 at the end.

Recommendations:

1. **Use your gesture** to assist your message when you are trying **to communicate an event**. It serves the dual purpose of providing information AND facilitating word retrieval (i.e. you are more likely to access the spoken verb when accurately imitating or replicating the action).

2. Whenever you are doing an activity try to **narrate** and **describe what you are doing**. The real-time actions should aid word retrieval and enable you to increase frequency of access to spoken verbs (and hopefully some short sentences).

3. **Always** try to **produce a sentence** when **describing something** that's happened – always use the 3 stage strategy to tell stories:
 1) who was there? where was it? when? 2) what was happening? 3)What happened next?

   Think of **who** is doing the action **and what** they are doing – try to start your sentences with the name of the person doing the action **OR** the pronoun (I, you, he, she, we, they etc.)

4. Try to **describe the positions of household objects** using sentences… i.e. "the bag is on the chair"). Try to use speech to give people instructions as much as possible.

5. Continue to **use WH questions** in conversations. Your understanding of each word's meaning (What / When / Where / Who / How / Why) has become more stable. Practise asking 2 or 3 questions in series changing the WH word to get a different piece of information.

6. **Try to speak with close family/friends on the telephone** or **video calling** as much as possible – you benefit from planning ahead of the call, anticipating information you might be asked and rehearsing key words (especially the greeting/opening sentence).

7. Practice **reading single words** that are 'regular' (i.e. spelled as they sound)

8. Continue to practise **reading short to medium length sentences** from **books, short newspaper articles** or written **texts** out loud – you benefit from support when reading (as follows):
   1. Read aloud - pointing to each word as you go
   2. Read aloud again and encourage pointing to the words as they are sounded.
   3. Read aloud again - both reading simultaneously
   4. For each line or sentence, state a word and ask person to find it
   5. For each line or sentence, point to a word and ask person to read it
   6. Read the passage aloud simultaneously

9. Consider updating the **CUESPEAK** app on your ipad – your local speech and language therapist will need to assist you by selecting appropriate tasks and calibrating the level of challenge. I would recommend that you prioritise exercises that focus on semantics (less everyday words among increasing numbers of closely related distracting options), verb naming/short sentence production and asking or answering 'wh' questions.
   You also have **TACTUS** on your ipad which has numerous exercises that will help – try to focus on spoken and written understanding (2 words or sentence to picture matching).

10. You have got better at opening your camera on your ipad and taking photos independently. Try to use your ipad regularly to **take photographs** of your **daily life** (things you see, places you go, people you see, food you eat, TV programmes you

watch, articles in the paper etc.)… try to learn how to **send these via Whatsapp** to selected family and friends to start conversations. You can also **use photographs to start conversations** in person – also try to retrieve and rehearse important words that you want to say when showing the picture.

11. Continue to use your **written scripts to help you write text messages to family –** you are really good at grouping and organising messages from the optional sentences and you are getting better at copying without making mistakes.

12. Use as many opportunities as possible to communicate with others. We have discussed

    seeking new communication opportunities and getting into new habits – **new groups** and routes to **increasing independence outdoors**.

I intend to speak with your local speech therapist to discuss next steps with therapy (building on the skills developed during the ICAP).

I am also **referring you to ……….**who provide weekly online groups for people with aphasia.

We look forward to meeting with you again at your review in 3 months currently scheduled for Monday ……………………… at 1pm

Best Wishes,

Speech and Language Therapist
Intensive Comprehensive Aphasia Programme

Cc:

Some helpful resources:

Psychological therapies

- Improving Access to Psychological Therapies (IAPT)

  If a patient is experiencing problems with their mood, they can be directed to their GP for advice and access to local support services. IAPT services provide free talking therapies to people with problems such as stress, anxiety or depression. Local services can be found here: https://www.nhs.uk/service-search/find-a-psychological-therapies-service/. They can self-refer or the GP can assist with a referral.

- Headspace - Easy to use app with guided mindfulness meditation for support with relaxation, managing stress and sleep: https://www.headspace.com/
- Calm - Easy to use app for sleep and meditation, including music, nature scenes and sounds for relaxation: https://www.calm.com/

Charities

- Headway (brain injury association)

  Headway is the UK-wide charity that works to improve life after brain injury by providing support and information services.

  Headway's range of e-booklets and factsheets cover many of the symptoms and practical issues associated with a brain injury, written for patients and families, and can be downloaded from: https://www.headway.org.uk/about-brain-injury/individuals/information-library/

- Stroke Association

  Provide specialist support for people affected by stroke including emotional support, communication support, exercise-based rehabilitation. Their local support services, stroke clubs and groups offer peer support to stroke survivors, carers, family and friends. Search for local services by postcode here: https://www.stroke.org.uk/finding-support/support-services

  Information on getting back to work after a stroke including a guide for employers: https://www.stroke.org.uk/life-after-stroke/getting-back-work

Volunteering

- Do It Organisation

  Volunteering can be a helpful way of gaining experience in a new area of work, building routine and work tolerance and developing skills. Information can be found at www.do-it.org on hundreds of online or face to face volunteering opportunities.

- The National Council for Voluntary Organisations

  Further information on volunteering and voluntary organisations can be found here: https://www.ncvo.org.uk/ncvo-volunteering/i-want-to-volunteer. Each locality has a Volunteer Centre where you can access advice about finding local volunteer roles.

Carer focused

- Carers Trust UK-wide network of local services providing information, advice, respite, advocacy and emotional support, including services for young carers too. https://carers.org/

- Carers UK - This charity campaigns for justice for carers. Their local branches provide a variety of support services, ranging from money advice and advocacy to emotional support and befriending https://www.carersuk.org/

B. Queen Square ICAP Equipment Inventory

1x Lenovo X390 Thinkpad

2x Apple ipads (equipped with Cuespeak, Tactus, Tactus Advanced, Listenin, Ireadmore etc.)

1x KS Boombar wifi speaker with soft case, USB charger and jack

2x Sennheiser 'over ears' headsets

2x Comprehensive Aphasia Tests (carry bags - each containing assessment booklet, disability questionnaire and manual)

1x Brain Injury Workbook (2nd edition) T. Powell – Speechmark

1x Colour-cards Sequences 6&8 step for adults
1x Colour-cards 'odd one out'
1x Colour-cards home

2x A3 whiteboards and 2 x A4 whiteboards
Assorted stationary
Assorted resources and plastic tray storage unit

C. Goal setting support tool

University College London Hospitals NHS
NHS Foundation Trust

Intensive Comprehensive Aphasia Programme @ Queen Square

1	2	3
a bit	quite	very

Name: Goal importance

Your goal (as you said it)	Therapy goal (as we see it)	Importance

Suggestions:

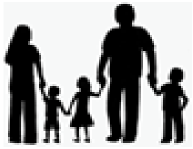
Speaking in groups. people

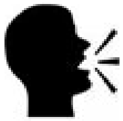
Speaking one to one.

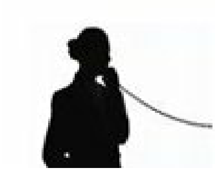
Using the phone

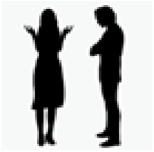
Understanding

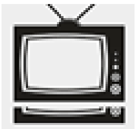
Understanding TV / films

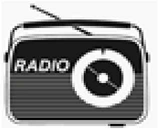

Understanding radio

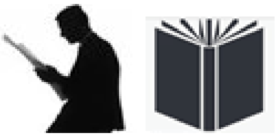
Reading newspaper / books

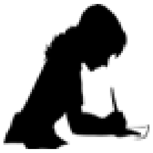
Writing

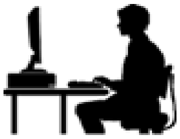
Using computers

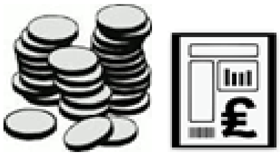

Using money / maths / managing bills

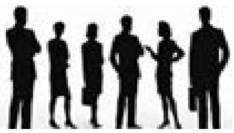
Getting back to work

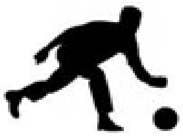
Getting back to activities

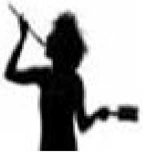
Following recipes

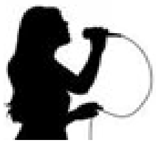
Karaoke / reading lyrics

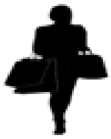
Online shopping

D. My aphasia and how to support me presentation

"My aphasia and how to support me"

Intensive Comprehensive Aphasia Programme @ Queen Square

1

What are the aims of this session?

To **discuss** the **frustrations** my **aphasia** can cause – for you and for me

To **discuss stroke and aphasia - help** you **understand it better**

To **discuss how everyone** has **adapted** to help **support** my **communication** – what's changed?

To **examine how some** of the well-intentioned **things you do to help me** can actually limit **recovery**

To **learn** new **ways** of **supporting my communication** to give me the **best chance** of **improving**

2

Introductions

- "introduce yourself"
- "explain how you know me"
- "what do you find challenging or frustrating?"
- "how do you support me to speak"

3

Stroke

Every stroke is **different** – the **location** and **size of** the **damage varies**

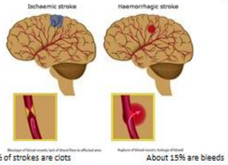

About 85% of strokes are clots   About 15% are bleeds

My stroke was a

The **brain areas for language** are usually **located** in the **left side** of the **brain** but **communicate with other areas** to perceive information, process it and plan how to respond

4

Aphasia

Language uses consistent **symbols** to **represent meanings**

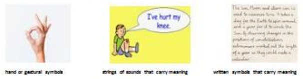

Aphasia is an acquired **problem** with **language**

People with aphasia lose some of their ability to understand and use the symbols of language that they have previously learned.

Different people with aphasia might have very different levels of ability in…

- *Understanding spoken words*
- *Understanding written words (reading)*
- *Speaking*
- *Writing*
- *Using gestural symbols*

5

Language can be used to **develop** and **refine** our **thinking**

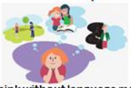

…but people **think <u>without</u> language** most of the time

The thoughts we turn into language (i.e. into symbols or a string of symbols) emerge from things we see, hear, smell, touch, feel, remember, want or need. They also emerge from different things experienced or considered in combination

When you were small (before you learned language) you **experienced things** and learnt about them **without knowing** the strings of **sounds or letters** that were used to represent them.

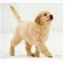

6

Think about the **picture** you just saw
Imagine a small child **thinking about this thing**

Remember – you don't have language… imagine how you feel… what do you notice with your senses?

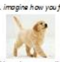

We continue to experience the world without language all our lives… learning symbols to express the meanings, feelings and associations that we identify

We notice similarities between things… and use language to show the difference

"pear"   "apple"

When we **experience something new**…

we use our **knowledge** of **similar things** to **build** an **idea** of what it might be

Only when we have seen it in its proper setting can we begin to understand it

… then we learn that it is called a "starfruit" *(in sounds and letters… in English)*

…and then we can refer back to it with other people that know what it is too.

7

The word **semantics** relates to **meaning**

Meaning is derived from properties, associations and the specificity of things we experience.

Properties (concepts) and associations (networks) exist beyond language…

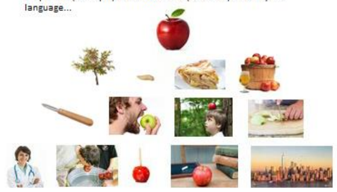

8

Our ability to perceive meaning relies on…

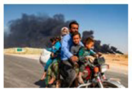

Intact semantics and problem solving essential to deductive reasoning:

Kuwait? Iraq? Syria?
1991? 2002? 2010?
burning oil?
motorbike – moving
family – overloaded bike
escaping danger?

Our ability to communicate the meaning relies on…

Good **planning/organisation** skills and reliable **access to at least 3 of the** required **words**

i.e. "escaping" "people" "motorbike"
OR
"the family are fleeing a dangerous situation on a motorbike"

9

Impaired semantics can sometimes **limit** a person's **interpretation** of **a situation** and recognition of its relevance to someone else

What is that? Is it supposed to be in the sandwich?

Is it dangerous?

Has the person I'm with seen it?

Should I tell them?

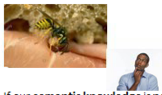

If our **semantic knowledge** is **poorly integrated** deductive reasoning can be restricted and **understanding another person's perspective** can be **impaired.**

10

Problems with **my language** are

Receptive (understanding speech / reading)

AND

Expressive (speaking / writing)

Think about the following sentence…

"pass me the orange from the table"

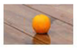

this sentence is easy to understand in the context of a single orange on a table… there is very little room for confusion. There are no other fruits or furniture items to create confusion and it is safe to assume that the sentence asks for the orange to be transferred even if the word 'pass' is difficult to understand.

The same sentence would be more difficult to understand in the following context.

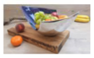

11

I have to **solve problems** <u>all the time</u> **to be confident** of my **understanding**

Context helps me with **problem solving**
(context can also refer to objects and actions that usually occur in/or before a given situation)

Sometimes context is **unclear** – this can happen when there are **too many** similar **options,** nothing typical has happened beforehand or the situation is not very familiar.

If I lack confidence in my understanding **I can** sometimes **stop responding** to information **or look** to others **for** confirmation or **help**

12

Neural plasticity

This term describes how neurones in the brain form connections that become more established as they are reinforced. These connections are influenced by the environment, interactions with people and also by intrinsic feedback.

Our brains change as we develop new routines, habits or skills – this is neural plasticity in action! Sometimes the newly forming routines or habits are good and sometimes they are bad

When somebody experiences a brain injury (such as a stroke) the existing connections are disrupted and new connections begin to form – it is important to try and ensure that these connections are desirable

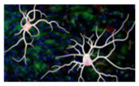

Note. Younger brains are more 'plastic' than older brains and can adapt / make new connections more easily
(remember that new connections are not always positive)

13

Unhelpful supportive behaviours

- Behaving differently towards me (because communication's difficult)
- Speaking first when opportunities to communicate arise
- Not providing enough 'communication opportunities' (i.e. messy hair, poorly applied lipstick, muddy floor)
- Excluding me from conversations
- Guessing or assuming what I'm trying to say
- Speaking for me when people ask me questions
- Speaking too quickly / providing too much information at once
- Using complicated grammar or phrasing
- Not using pointing/gesture or props to 'show what you mean'
- Not using reducing sound cues or mouth shapes to help me say the target word
- Not encouraging me to order for myself in restaurants / cafes
- Other

14

Helpful supportive behaviours

- Try to treat me normally – give me space to plan responses and initiate questions of my own
- When something unusual is present (or has happened) try to support my recognition. Do this subtly if you can using eye contact (looking at the 'stimulus') to help me see the opportunity
- Encourage other people (i.e. professionals / shop-assistants) to speak to me and try not to speak for me (give me space and/or support I need to achieve even a single word response or show what I want)
- Include me in group discussions but try to speak one at time and point to the person you are addressing (it helps me follow the conversation)
- Use shorter, simpler phrasing when you remember and avoid complicated grammar – use pointing, gesture and props!
- Use subtitles on the TV – they increase confidence in my understanding
- Try rephrasing (saying the same thing differently) and check my understanding
- Use the initial sounds of words (or mouth shapes) to 'cue' me
- Encourage me to 'talk around a word I can't find' – "describe it" "give clues"
- Encourage me to try challenging communication (e.g. ordering for myself, speaking on the telephone, reading aloud from the paper) with strategies

15

Important

All the **unhelpful supportive behaviours** listed **are a product** of **your** neuroplastic **adaptation** to **my change in ability**

They are very **normal** and evolve **because you are trying to help** me

Very **quickly** after my stroke **I became reliant on** some of the **'unhelpful support'** in **situations** where I felt **challenged or under-confident**

I need you to try and **change** the way you support me **to help** develop **change in my communication** and to reinforce emerging abilities

<u>**This requires** neuroplastic **changes in your brains too**!</u>

16

E. 2022 CLINICAL REFLECTIONS

(advice & clinician suggested changes to ICAP)

- Ensure flexibility and reflexivity within the treating team
- Preserve experienced SLT and Psychology staffing (the programme relies on high levels of shared knowledge and trust between clinicians)
- Continually refine timetabling and the content of the programme in response to stakeholders' feedback
- Maintain and continually refine an efficient documentation system
- Pursue secure bookings of largely consistent clinic rooms to reduce stress associated with set-up, resource access/availability and participant experience)
- Secure daily group room access
- Improve organisation and access to regularly used therapy resources (secure storage options)
- Consider a weekly remote ICAP group session each week - former participants could join current participants for a review session focussed on weekly objectives and recent achievements as well as engaging in group communication activities. Supporting current participants to login and join on their phones (as independently as possible at a set time) would help establish the routine of engagement. Maintaining a regular remote link to former participants could help support their transition back to everyday living and assist with goal achievement (via increasing accountability). Such an addition would help reduce 'cliff edge' transition.
- Enhance manual instructions for CAT spoken picture description facilitation and scoring (to ensure
- Schedule and protect daily administration time for all clinicians
- Recognise the benefits of well-matched participants engaging in paired therapy
- Improved feedback systems so that participants and their carers can shape the ICAP
- Increase use of volunteers (etc. supporting mornings, breaks and lunchtimes)
- Introduce a network of buddy systems for post-ICAP conversation practice (facetime/whatsapp/email 'pen pals') matching participants from different cohorts based on compatibility factors (age/interests etc.) and potentially in-person buddy systems for people living close to one another. Such systems could be peer maintained beyond introduction.
- Modify the keyworker system to increase 'whole team' focus on all cohort participants – introduce deputy key clients to ensure continuity of focus during periods of leave
- Integration of 'aphasia champions' – previous ICAP attendees talking about living well with aphasia
- Integration of 'carer champions' – carers of previous attendees talking about making positive changes
